# Overexpression of the Endosomal Anion/Proton Exchanger ClC-5 Increases Cell Susceptibility toward *Clostridium difficile* Toxins TcdA and TcdB

**DOI:** 10.3389/fcimb.2017.00067

**Published:** 2017-03-13

**Authors:** Frederike Ruhe, Alexandra Olling, Rasmus Abromeit, Dennis Rataj, Matthias Grieschat, Andre Zeug, Ralf Gerhard, Alexi Alekov

**Affiliations:** ^1^Institute for Neurophysiology, Hannover Medical SchoolHannover, Germany; ^2^Institute for Toxicology, Hannover Medical SchoolHannover, Germany

**Keywords:** *C. difficile*, TcdA, TcdB, ClC-5, CLC transport, CLC proteins, endocytosis, endosomal acidification

## Abstract

Virulent *C. difficile* toxins TcdA and TcdB invade host intestinal epithelia by endocytosis and use the acidic environment of intracellular vesicles for further processing and activation. We investigated the role of ClC-5, a chloride/proton exchanger expressed in the endosomes of gastrointestinal epithelial cells, in the activation and processing of *C. difficile* toxins. Enhanced intoxication by TcdA and TcdB was observed in cells expressing ClC-5 but not ClC-4, another chloride/proton exchanger with similar function but different localization. In accordance with the established physiological function of ClC-5, its expression lowered the endosomal pH in HEK293T cells by approximately 0.6 units and enhanced approximately 5-fold the internalization of TcdA. In colon HT29 cells, 34% of internalized TcdA localized to ClC-5-containing vesicles defined by colocalization with Rab5, Rab4a, and Rab7 as early and early-to-late of endosomes but not as Rab11-containing recycling endosomes. Impairing the cellular uptake of TcdA by deleting the toxin CROPs domain did not abolish the effects of ClC-5. In addition, the transport-incompetent mutant ClC-5 E268Q similarly enhanced both endosomal acidification and intoxication by TcdA but facilitated the internalization of the toxin to a lower extent. These data suggest that ClC-5 enhances the cytotoxic action of *C. difficile* toxins by accelerating the acidification and maturation of vesicles of the early and early-to-late endosomal system. The dispensable role of electrogenic ion transport suggests that the voltage-dependent nonlinear capacitances of mammalian CLC transporters serve important physiological functions. Our data shed light on the intersection between the endocytotic cascade of host epithelial cells and the internalization pathway of the large virulence *C. difficile* toxins. Identifying ClC-5 as a potential specific host ion transporter hijacked by toxins produced by pathogenic bacteria widens the horizon of possibilities for novel therapies of life-threatening gastrointestinal infections.

## Introduction

The spore-producing gram-positive bacterium *Clostridium difficile* (*C. difficile*) is one of the leading causes of healthcare-related infections. The symptoms of *C. difficile* infections (CDI) range from light to very severe and life-threatening antibiotic-associated diarrhea and pseudomembranous colitis. *C. difficile* bacteria produce two main virulence proteins, the large glucosyltransferases Toxin A (TcdA) and Toxin B (TcdB). These toxins play a central role in the development of the bacterial pathogenicity at the cellular level and of the clinical symptoms at the whole organism level. (Voth and Ballard, 2005) The major cytotoxic effects of TcdA and TcdB develop through a cascade of events that can be divided into three major steps: (a) binding, (b) endocytosis, and (c) translocation and release of the toxin's N-terminus from the endosomes into the host cytosol (Tucker and Wilkins, 1991; Jank et al., 2007; Papatheodorou et al., 2010). The activated toxin N-termini produced in the last step inactivate members of the Ras superfamily of small GTPases via glucosylation (Pfeifer et al., 2003; Just and Gerhard, 2005; Jank et al., 2007; Pruitt et al., 2010). Toxin-mediated inactivation of the small GTPases leads to disorganization of the cytoskeleton and changes in cell morphology, often denoted as cell rounding (Just et al., 1995; Nottrott et al., 2007). This particular step is relatively well described and represents one of the major mechanisms underlying the cytopathic effects of TcdA and TcdB. The preceding events have been also intensively investigated. It is known that at least two host receptor proteins support toxin attachment to the surface membrane of attacked cells (LaFrance et al., 2015; Yuan et al., 2015). The subsequent internalization includes (but is not restricted to) the clathrin-mediated endocytosis (CME) pathway (Papatheodorou et al., 2010; Gerhard et al., 2013; Chandrasekaran et al., 2016). Importantly, V-ATPase-dependent acidification of endocytotic vesicles seems to be essential for the following cytotoxic effects; it triggers significant conformational changes of TcdA and TcdB that lead to the formation of channels in the vesicle's membrane and allow the toxin N-termini to access the cytosol (Barth et al., 2001; Giesemann et al., 2006; Schwan et al., 2011).

In light of the permissive role of vesicular acidity for the cytopathic action of bacterial toxins, we set out to investigate the potential involvement of the human Cl^−^/H^+^ exchanger ClC-5 in the processing and activation of *C. difficile* TcdA and TcdB. The choice was motivated by the importance of ClC-5 for the processes of endocytosis and endosomal acidification (see for a review Jentsch, 2008). ClC-5 is a Cl^−^/H^+^ exchanger (Picollo and Pusch, 2005; Scheel et al., 2005) that is expressed and physiologically active in cells constituting the gastrointestinal epithelial barrier attacked by *C. difficile* toxins. Specifically, ClC-5 has been found in early and early-to-late endosomes in rat intestinal epithelial cells (Vandewalle et al., 2001). In gastric parietal cells, ClC-5 has been shown to associate with the H^+^/K^+^-ATPase and to increase the activity of gastric proton pumps (Takahashi et al., 2014). The physiological role of ClC-5 has been controversially discussed. There is evidence that it provides counter-ions to enhance the acidification of endosomes, a process that is actively driven by the vesicular proton pumps (V-ATPases) (Lloyd et al., 1996; Piwon et al., 2000; Wang et al., 2000). However, it has been also proposed that ClC-5 can directly acidify endosomes by its Cl^−^/H^+^ exchange function (Smith and Lippiat, 2010a). The principles of operation of these proteins have been also extensively investigated and it is known that the CLC transport activity critically depends on two glutamate residues, the so-called gating glutamate at position E211 (Picollo et al., 2010) and the so-called proton glutamate at position E268 (Zdebik et al., 2008).

It is important to note that increased severity and higher mortality rates are associated with CDI in patients with inflammatory bowel disease (IBD) (Issa et al., 2008). Remarkably, increased expression of ClC-5 has been detected in IBD patients and in mice with experimentally induced colitis. *Vice versa*, increased susceptibility to dextran sulfate sodium-induced colitis was found in *Clcn5* knockout mice. (Sullivan et al., 2009; Alex et al., 2010) These data additionally support the notion that human ClC-5 might be involved in the development of *C. difficile*-induced cytotoxicity and the pathophysiology of CDI. To test this hypothesis, we expressed human ClC-5 in HEK293 and colon HT29 cells and elucidated the effects of this manipulation on the endocytosis and cellular effects of TcdA and TcdB.

## Results

### ClC-5 expression increases susceptibility to intoxication by TcdA and TcdB in an acidification-dependent manner

**Figure 1:**
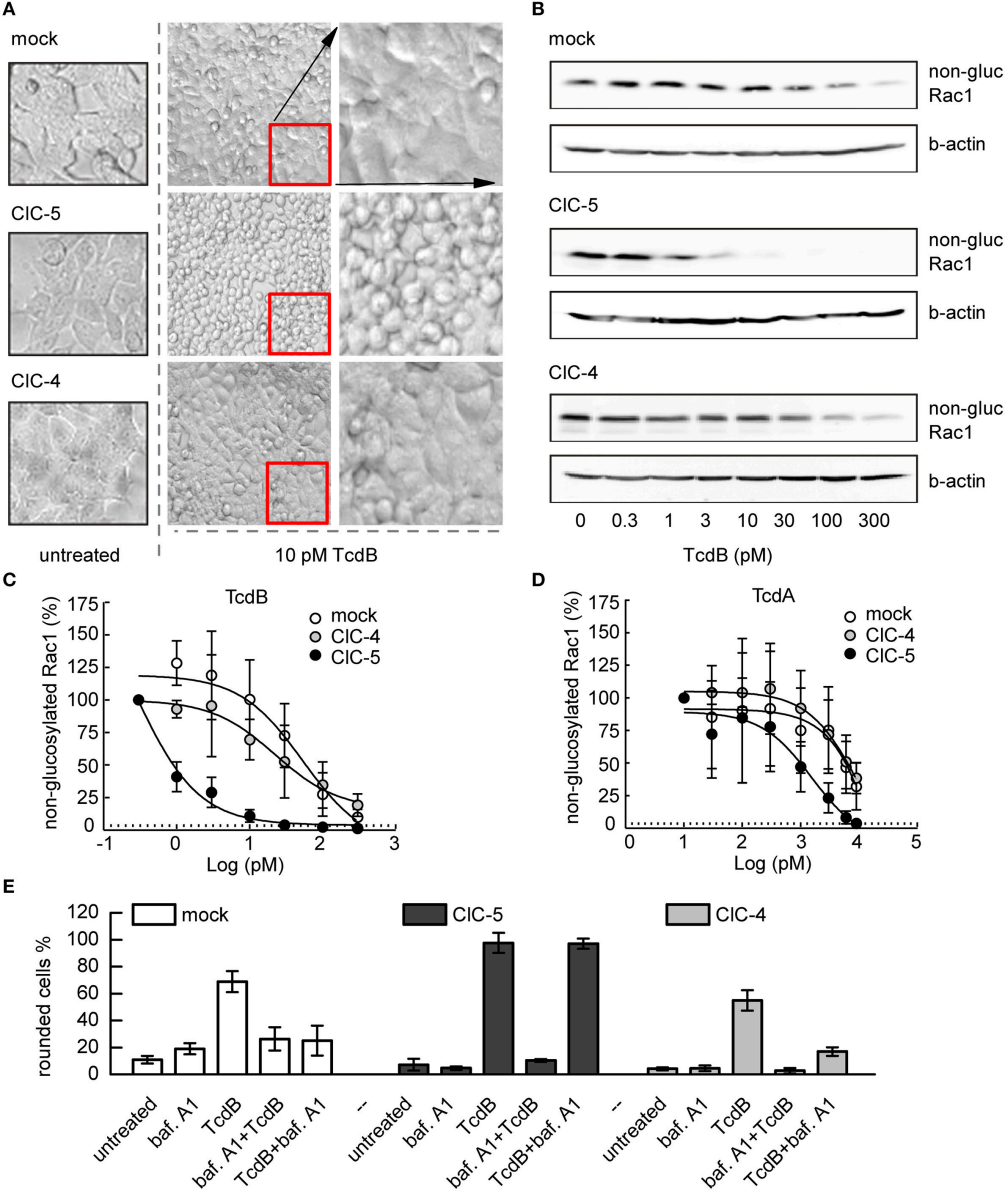
**ClC-5 expression increases the susceptibility of HEK293 cells toward TcdA and TcdB. (A)** HEK293 cells stably transfected with ClC-5-mCherry, ClC-4, or mCherry alone (mock) treated with 10 pM TcdB for 1 h at 37°C. The micrographs show the cell morphology of healthy untreated cells (left), as well as cell rounding induced by toxin-catalyzed glucosylation of the Rac1 GTPase (middle). Enlarged micrograph sections of the toxin-treated cells are depicted in the right lane. **(B)** Representative concentration-dependent analysis of Rac1 glucosylation by TcdB. Glucosylation was determined by immunoblotting using glucosylation-sensitive anti-Rac1 antibody. A decrease of the anti-Rac1 signal (non-gluc Rac1) reports an increase of the glucosylation of Rac1. Beta-actin is used as a control for protein load. **(C,D)** Densitometry evaluation of immunoblots as depicted in **(B)** showing Rac1 glucosylation by TcdB and TcdA (*n* = 3, mean ± SD). **(E)** Effects of V-ATPase inhibition by bafilomycin A1 (baf. A1) on cell rounding induced by toxin-catalyzed glucosylation of Rac1 GTPase. Examined were HEK293 cells stably transfected with mCherry (mock), ClC-4, or mCherry-tagged ClC-5. Bars represent the percentage of rounded cells at the end of the experiment. Controls were either treated with baf. A1 (baf. A1) or with 2 nM toxin (TcdB). In the first combination experiment, cells were first treated with baf. A1 for 10 min before the toxin (2 nM) was added to show the maximum inhibitory effect (baf A1 + TcdB). For limited uptake of TcdB, baf. A1 was added 10 min after addition of 2 nM toxin (TcdB + baf. A1). Cell rounding was examined by light microscopy at the end of the experiment (total time 100 min at 37°C, *n* = 3, mean ± SD).

We first investigated the cytotoxic effects of the more potent *C. difficile* toxin, TcdB, on HEK293 cells stably expressing ClC-5 (Figure 1). The TcdB-induced disruption of the actin cytoskeleton is readily observed as changes in the form and morphology of the affected cells (Just et al., 1995; Nottrott et al., 2007). Figure 1A shows representative images of untreated cells and cells intoxicated with 10 pM TcdB. Control cells stably transfected with the fluorescent protein mCherry were almost unaffected by the applied toxin concentration. In contrast, a significant percentage of the cells expressing ClC-5 exhibited roundish form (Figure 1A, see also Supplementary Figure S1 for effects on colon HT29 cells). We also applied TcdB on cells stably expressing ClC-4. ClC-4 is relevant for the central nervous system; it is closely related to ClC-5 but mainly found in membranes of the endoplasmic reticulum (Okkenhaug et al., 2006; Veeramah et al., 2013; Hu et al., 2016). At the used TcdB concentration, ClC-4-expressing cells behaved similarly as the mCherry-expressing cells, suggesting that the role of ClC-5 is specific.

The action of *C. difficile* toxins can be conveniently observed in the time domain by monitoring the transepithelial resistance (TER) of confluent epithelial cell layers (Gerhard et al., 1998). In particular, the TER is defined by the barrier integrity of epithelia with tight junctions. A decrease of the TER indicates disruption of the barrier function and an increase in epithelial paracellular permeability. Applying the method on Madin-Darby canine kidney cells (MDCK II) cultivated to confluency on filter inserts showed that the TER decreases faster in ClC-5 expressing cells exposed to TcdA (Supplementary Figure S2A). For example, 1 h of basolateral toxin application decreased the TER of ClC-5-expressing cells by approximately 50%. In contrast, a comparable TER decrease was observed in untransfected cells after 3 h of TcdA incubation. These effects additionally demonstrate the role of ClC-5 for the accelerated intoxication by TcdA and indicate that this ion transporter stimulates the uptake or translocation of *C. difficile* toxins.

We next investigated the effects of TcdA and TcdB on Rac1 glucosylation, the primary cause for the following cytoskeleton disruption and cell rounding (Just and Gerhard, 2005). Immunoblotting with a glucosylation-sensitive anti-Rac1 antibody (Genth et al., 2006) showed increased glucosylation in toxin-exposed ClC-5-expressing cells when compared to mock cells (expressing mCherry). Rac1 glucosylation in ClC-5-expressing cells was also increased when compared to the effect in cells stably transfected with ClC-4 (decrease of the anti-Rac1 antibody signal, Figures 1B–D). To additionally demonstrate that the intoxication specifically shifts the equilibrium between glucosylated and non-glucosylated Rac1, we also tested how toxin treatment affects the total amount of Rac (Supplementary Figure S2B). Please note that the differences in the intensity of the Rac1 bands shown in Figure 1 and Supplementary Figure S2 are due to differences of loaded protein amounts and Western Blot detection.

Based on the role of ClC-5 for vesicular acidification (Jentsch, 2008), we conducted additional experiments in which we applied the specific V-ATPase blocker bafilomycin A1 (Yoshimori et al., 1991). High concentration of TcdB (2 nM) was used in these experiments to maximize toxin uptake. The ATP-dependent acidification was blocked either 10 min before the application of TcdB to prevent toxin translocation, or 10 min after toxin application to allow limited translocation. The cytopathic cell rounding was assessed after an additional 90-min incubation at 37°C. In the first 10 min, cells expressing mCherry or ClC-4 showed no visible cell rounding which indicates poor toxin uptake or translocation (Figure 1E). In contrast, a significant number of cells expressing ClC-5 become rounded. It is established that bafilomycin interferes mainly with the pH-dependent early-to-late endosome and the late-endosome-to-lysosome maturation and not with the preceding endocytosis steps (Yoshimori et al., 1991; Bayer et al., 1998). These experiments suggest, therefore, that enhanced rates of endocytosis and early endosome acidification and maturation might be decisive for the increased cytopathic effects of large *C. difficile toxins* in ClC-5 expressing cells.

### The cytopathic effect of TcdA is reduced by knockdown of CLC-5

**Figure 2:**
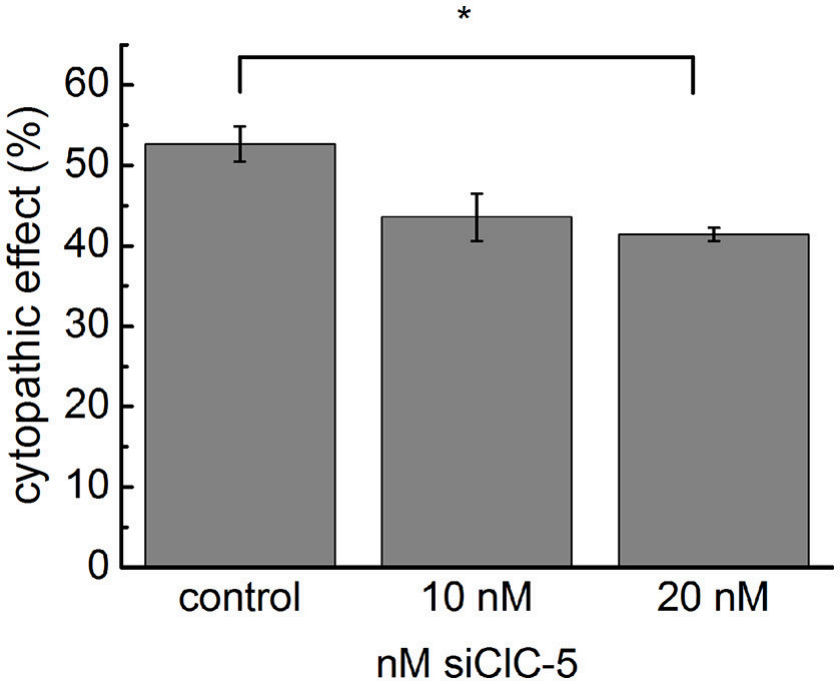
**Downregulation of endogenous ClC-5 decreases TcdA cytotoxicity**. The expression of ClC-5 in HT29 cells was reduced by transfection with siRNA targeting human ClC-5 (siClC-5). Transfection with non-targeting RNA was used as a control. Bars represent the quantitative analysis of the cytotoxic action of 3 nM TcdA (percentage of roundish cells, See Supplementary Figure S3). Data are summarized as means ± SEM (*n* = 3 for the control and *n* = 7, *n* = 7 for siClC-5 treated cells). ^*^Indicates a statistical difference; *p* < 0.05.

To provide evidence for the biological relevance of the accelerated intoxication associated with ClC-5 overexpression, we used small interference RNA (siRNA) to reduce the endogenous expression of ClC-5 in colon HT29 cells. The effectiveness of the method to reduce the number of expressed ClC-5 proteins was tested on HEK293 cells stably expressing mCherry-tagged ClC-5. In these experiments, the number of ClC-5 proteins (measured by the mCherry fluorescence intensity) was clearly reduced 72 h after the transfection (Supplementary Figure S3A). Similarly, quantitative RT-PCR demonstrated a relative downregulation of endogenously expressed ClC-5 by approximately 50–70% in HT29 cells (Supplementary Figure S3B). In the following experiments, we intoxicated HT29 cells with 3 nM TcdA and assessed the associated cytopathic effects by quantifying the percentage of rounded cells. To differentiate between transfected and untransfected cells, a plasmid containing the sequence of the human CD8 antigen was cotransfected together with the siRNA. Commercial microbeads coated with anti-CD8 antibodies were added to the cell/toxin mixture and used to visually select transfected cells (see Supplementary Figure S3C). The downregulation of ClC-5 in these experiments reduced the toxin potency on HT29 cells by approximately 10% (Figure 2). The significant reduction of the toxin potency demonstrates the physiological relevance of the ClC-5-mediated effects and the potential therapeutic importance of altering ClC-5 activity in gastrointestinal cells. The incomplete block of the cytopathic effects is not surprising. On the one hand, ClC-5 expression could not be completely blocked by the procedure; on the other hand, the cellular V-ATPases which are the major force behind vesicular acidification are expected to be active in the treated cells. It is arguable that the transfection efficiency of plasmid DNA and siRNA is not identical and therefore that the cells expression CD8 antigen are not necessarily also transfected with siRNA. The RNA downregulation of up to 70% indicates, however, that more cells are transfected with siRNA than with the pLeu2-CD8 plasmid (which showed a transfection efficiency of around 30%). This increases the probability that the pleu2-CD8 expressing cells were also transfected with siRNA.

### TcdA colocalizes with CLC-5 in vesicular compartments

**Figure 3:**
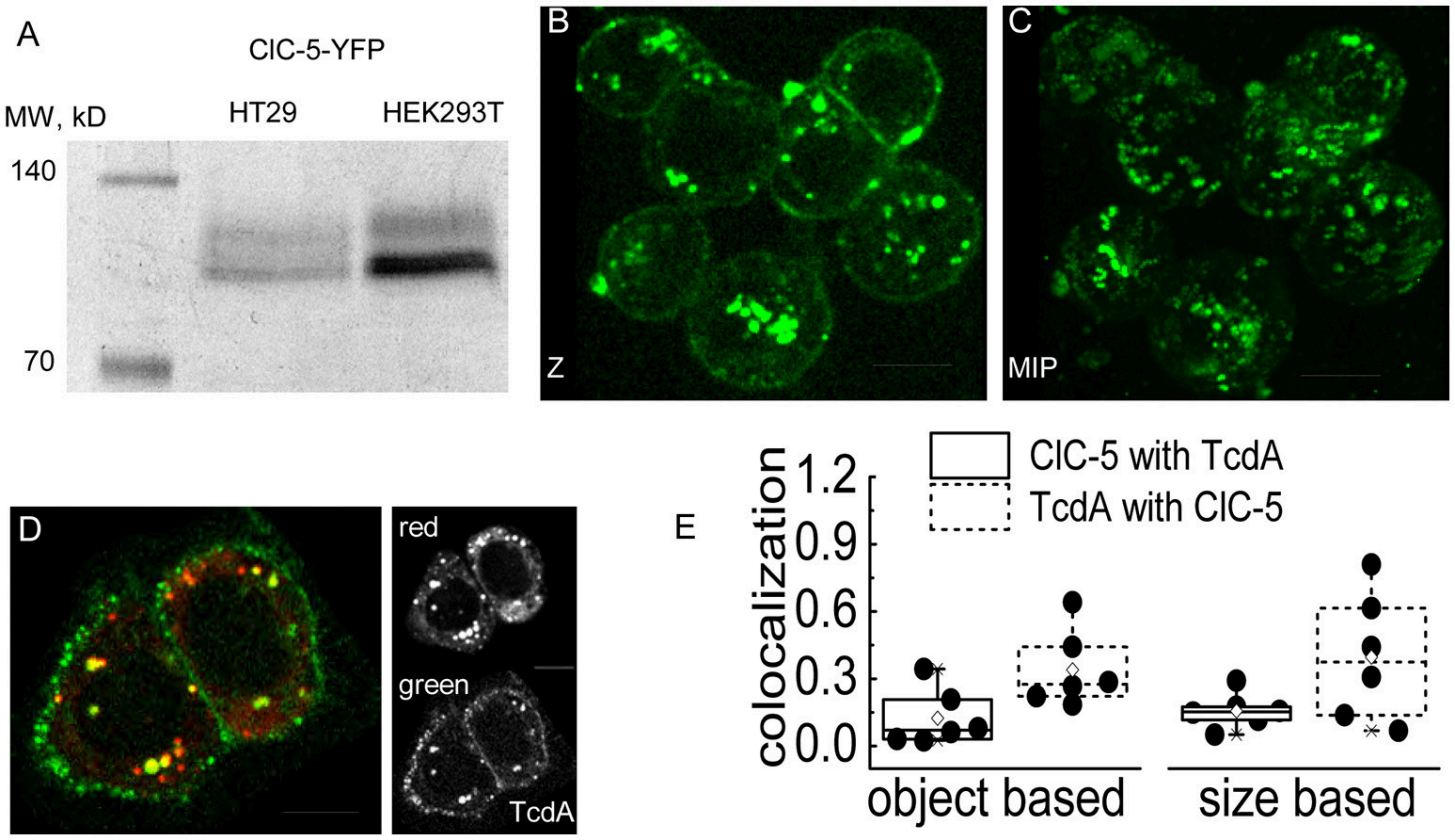
**Colocalization of TcdA-EGFP and ClC-5 in living cells. (A)** Electrophoresis SDS-PAGE of ClC-5-EYFP. Whole cell lysates of HT29 and HEK293T cells transiently expressing ClC-5-EYFP were separated by SDS-PAGE electrophoresis (60 μg of HT29 and 30 μg of HEK293T whole protein lysate). The first lane shows a standardized fluorescent marker with defined molecular weights of the bands indicated in kDa. **(B,C)** Representative live-cell confocal images (Z- single Z-section; MIP-maximum intensity projection) of HT29 cells after internalizing TcdA-EGFP for intervals ranging from 6 to 15 min at room temperature (RT). **(D)** Representative live-cell dual color confocal image showing the localization of TcdA-EGFP and ClC-5-mCherry in HT29 cells. Cells transiently expressing mCherry-tagged ClC-5 (red) were incubated with TcdA-EGFP (green) for an interval of 6–10 min at RT. All images were obtained using a spinning disc confocal microscope. **(E)** Box plots summarizing the results from an object- (left) and size-based (right) colocalization analysis of images as depicted in **(D)** corresponding to the number of overlapping objects and the overlapping areas of the objects identified as ClC-5- and TcdA-containing objects. Six cells were analyzed [single cells (•), 25–75% (☐, large box), mean (♢), median (—) as well as 99and 1%, respectively (X)]. Scale bars correspond to 10 μm.

A mechanism in which endocytosis and endosomal acidification enhanced by ClC-5 leads to faster delivery of the enzymatically active toxin N-terminal domain requires the toxins to localize in vesicles containing this transporter at least during the initial internalization phase. To test this hypothesis, we performed live cell imaging experiments in human colon cancer cells (cell line HT29) that are often used as a model for the colon epithelial cells attacked by *C. difficile*. The transporter was labeled with a fluorescent tag (mCherry or EYFP) covalently fused to its carboxy-terminus (Grieschat and Alekov, 2012). TcdA was visualized using an EGFP fluorescent tag inserted between the intermediate translocation domain and the C-terminal combined repetitive oligopeptides (CROPs) domain of the toxin (Olling et al., 2011). SDS-PAGE analysis confirmed that HT29 cells express and correctly process transfected ClC-5 (Figure 3A). Two distinct bands with the expected molecular weight of around 90 kDa were visible. In accordance with the established glycosylation of ClC-5 transporter (Jouret et al., 2004), the upper band represents complex-glycosylated and the lower band non-glycosylated and core-glycosylated ClC-5. The toxicity of TcdA was enhanced in these cells upon transfection with ClC-5 (Supplementary Figure S1). Upon incubation, fluorescent EGFP-tagged TcdA was detected at the plasma membrane and in intracellular vesicles in both untransfected and in ClC-5-expressing HT29 cells (Figures 3B–D). ClC-5 was localized in intracellular vesicles and a significant colocalization with internalized toxin was visible (Figure 3D). In contrast, expression of fluorescently labeled ClC-4 led to staining of the endoplasmic reticulum and showed no specific localization with internalized TcdA-EGFP (Supplementary Figure S4). The lower expression levels of ClC-5 in transfected HT29 cells (Figure 3D, inset “red”) seem to reflect the fact that this cell line is not optimized for the processing of plasmid DNA as is for example the HEK293T cell line.

We next quantified the colocalization between ClC-5 and TcdA using the Fiji plugin SQUASSH which is superior for analyzing “asymmetric” data, i.e., data for which many of the objects containing one of the investigated markers colocalize with objects containing the second marker but not *vice versa*. The analysis is superior also for noisy data (Rizk et al., 2014). We reasoned that the criteria fit our images since we did not expect a perfect overlap between the labeled vesicles in the two fluorescence channels. In particular, it appears unlikely that TcdA saturates all of the ClC-5-containing vesicles. Moreover, the weak fluorescence of internalized TcdA-GFP leads to relatively high noise in the corresponding imaging channel. The workflow of the analysis is exemplarily presented in Supplementary Figure S5. The object-based analysis showed that approximately 12% of the objects identified in the ClC-5 channel colocalized with TcdA objects (colocalization of 0.12; theoretical minimum–0 for no colocalization, theoretical maximum–1 for perfect colocalization); *vice versa*, approximately 34% of the objects identified in the TcdA channel colocalized with ClC-5 objects (Figure 3E, colocalization of 0.34). Using the same plugin, we also performed size-based analysis calculating the relative volume of the colocalizing pixels normalized to the full volume of all identified objects in each channel. The values obtained in this way were similar to the ones obtained by the object-based strategy: approximately 16% of the ClC-5 pixels colocalized with TcdA and approximately 40% of the TcdA pixels colocalized with ClC-5 (Figure 3E).

We also attempted to quantify time-dependent changes of the colocalization between both fluorophores. After a binding step with TcdA-EGFP at 4°C, cells were incubated for 0, 8, or 15 min at 37°C to allow internalization of the toxin. At the specified times, cells were fixed with methanol or paraformaldehyde to stop the processing of internalized toxins. Surprisingly, independent on the type of the fixation procedure, we detected only very weak EGFP fluorescence in intracellular vesicles (Supplementary Figure S6). These results resemble the findings of Kern and Feig (2011) who also reported a low detection of intracellular EGFP-tagged TcdA. The live-cell experiments (Figure 3) suggest, however, that a failure to internalize is not responsible for these effects. This conclusion is also supported by the fact that cell rounding induced by TcdA-EGFP occurs to a similar extent as if induced by wild type TcdA, i.e., that the cytotoxicity of the tagged toxin is preserved. Therefore, the encountered low number of intracellular vesicle-like spots in these experiments seems to result from fluorophore bleaching due to the applied fixation procedures. Significant negative effects of fixatives on the EGFP fluorophore have been reported by others (Brock et al., 1999; Billinton and Knight, 2001). In our case, the bleaching/quenching of internalized EGFP might have been further enhanced by the low vesicular pH of the endosomes.

### Transport deficient ClC-5 enhances cytopathic action of TcdA similarly as the transport-competent variant

**Figure 4:**
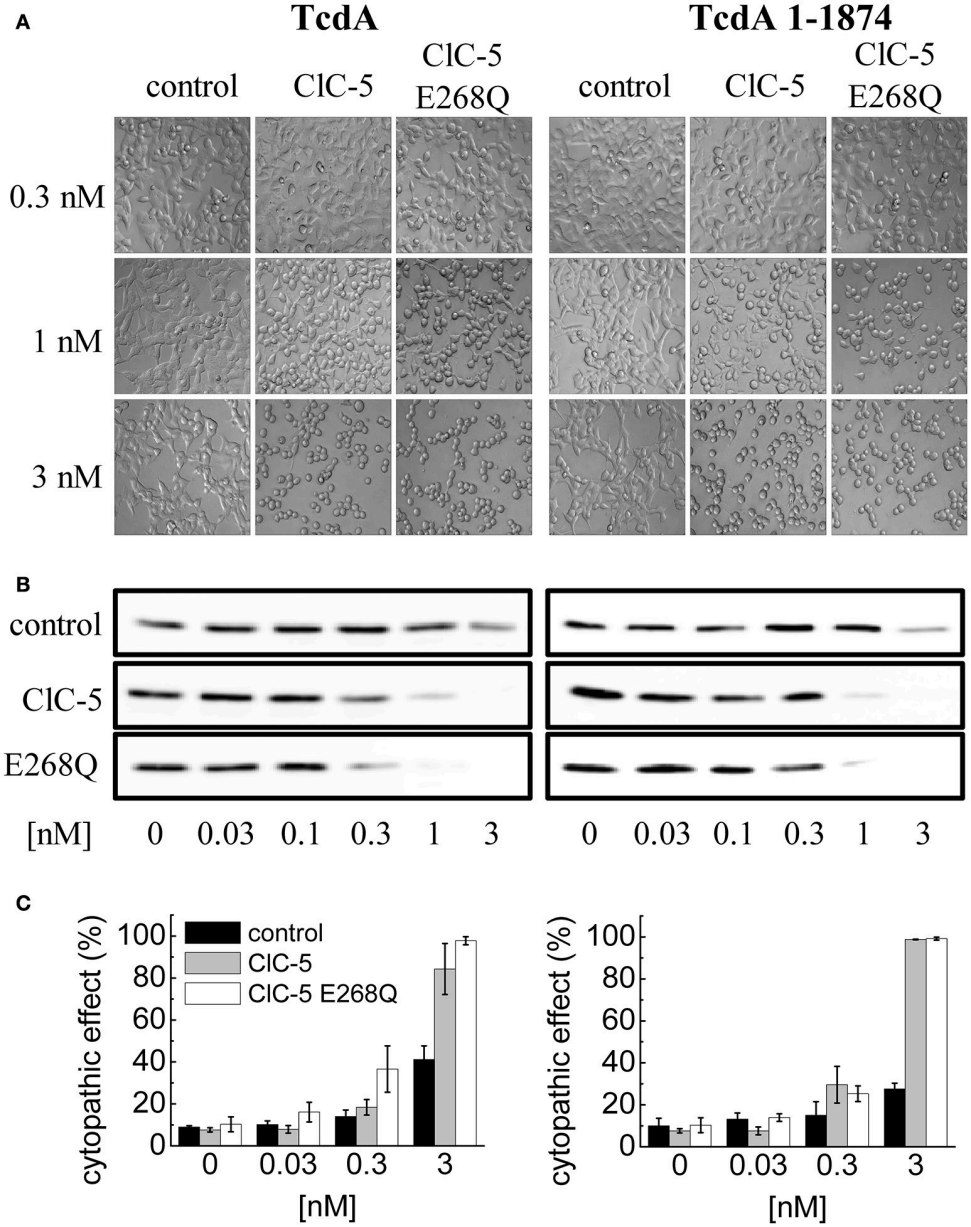
**Increased TcdA susceptibility of ClC-5 transfected cells is independent of electrogenic H^+^/Cl^−^ antiport. (A)** HEK293 cells stably transfected with the fluorescent mCherry (mock), ClC-5-mCherry and ClC-5-mCherry E268Q were treated with 0.3, 1, and 3 nM of full length TcdA (left) or CROPs-truncated (right) TcdA 1-1874. The micrographs depict cell rounding induced by toxin-catalyzed glucosylation of Rac1 GTPases. **(B)** Representative concentration-dependent analysis of Rac1 glucosylation by TcdA or TcdA 1-1874. Glucosylation kinetics was determined by immunoblotting using glucosylation sensitive anti-Rac1 antibody. A decrease of the Rac1 signal (non-gluc Rac1) correlates with the extent of glucosylation. **(C)** Concentration-dependent cytopathic effect of TcdA or TcdA 1-1874 on ClC-5- or ClC-5 E268Q expressing cells, measured by calculating the proportion of roundish cells to cells with healthy morphology.

Next, we set out to elucidate the impact of the ClC-5-mediated electrogenic ion transport for the enhanced action of *C. difficile* toxins. Since no potent pharmacological blockers for ClC-5 are available, we decided to use point mutation E268Q. Glutamate E268 is positioned near the intracellular protein-water interface and forms the entry of the separate proton transport pathway in CLC transporters. This highly conserved glutamate serves as an intracellular H^+^-acceptor and is obligatory required for the coupled exchange of chloride and protons (Accardi et al., 2005). Neutralizing the electric charge of this glutamate in the bacterial EcCLC leads to uncoupling of the CLC transport and allows anions to be transported without the antiport of protons (Accardi et al., 2005). Analogous charge neutralizations in mammalian CLC exchangers abolish not only proton but also anion transport (Zdebik et al., 2008; Smith and Lippiat, 2010b; Grieschat and Alekov, 2012). In other words, transfecting mutation E268Q results in the expression of a silent ClC-5 protein that does not mediate electrogenic ion transport. Surprisingly, however, overexpression of this mutant was also able to increase the cytopathic action of TcdA (Figures 4A–C). We hypothesized, therefore, that mutant E268Q might similarly as WT ClC-5 enhance the endocytotic toxin uptake. The internalization in this case might utilize different endocytosis pathway and the underlying process might not obligatory depend on the electrogenic transport function of the protein. To test this hypothesis, we investigated the effects of a truncated TcdA peptide in which the domain containing the so-called combined repetitive oligopeptides (CROPs) is removed (Papatheodorou et al., 2010; Olling et al., 2011; Gerhard et al., 2013). This deletion greatly reduces toxin uptake and the modified peptide seems to be internalized by a pathway that differs from the one followed by WT TcdA (Gerhard et al., 2013). Its application allows testing whether the toxin internalization proceeds more effectively along this alternative pathway in cells expressing ClC-5 E268Q. However, intoxication induced by the CROPs-truncated TcdA (TcdA 1-1874) was similarly enhanced by both ClC-5 WT and ClC-5 E268Q (Figures 4A–C, right). ClC-5 WT and E268Q seem, therefore, not to enhance different endocytosis pathways.

**Figure 5:**
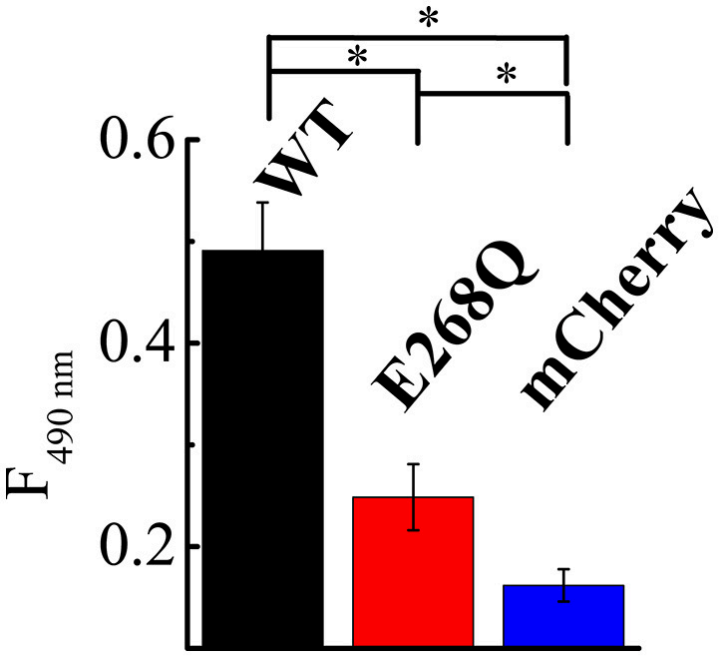
**ClC-5 and ClC-5 E268Q enhance the internalization of TcdA**. HEK293 cells stably transfected with the fluorescent mCherry (blue), ClC-5-mCherry (WT, black) and ClC-5-mCherry E268Q (red) were treated for 45 min on ice followed by a 5-min incubation at 37°C with 200 nM TcdA labeled with an Atto425. TcdA uptake was assessed by the Atto425 fluorescence intensity excited at 425 nm and measured at 440–650 nm. To correct for the different number of cells in the individual experiments, cell density was additionally measured using by absorbance spectrophotometry (Supplementary Figure S5). 9 Petri dishes were analyzed for ClC-5 WT and E268Q and 10 for mCherry on two different days. Bars represent means ±SEM at 490 nm emission. ^*^ Indicates a significant difference between two data sets. The significance was tested using a two-tailed *t*-test and significance was set at a *P*-value of <0.05.

To directly compare the effects of both ClC-5 constructs on endocytosis, we quantified the amount of toxin that is bound and internalized during a 5-min incubation at 37°C. The experiment showed that whereas both ClC-5 WT and ClC-5 E268Q increase the uptake of TcdA, the transport-deficient mutant is half as effective in doing so (Figure 5, Supplementary Figure S7; approximately 5-fold increase for ClC-5 WT vs. approximately 2.5-fold for ClC-5 E268Q). This effect might be explained, for example, by postulating that mutant E268Q ClC-5 is less effective in enhancing the overall rate of endocytosis. Unfortunately, there is no mouse model for the E268Q mutant, but mice expressing the uncoupling mutation of the proton glutamate E211A show impaired renal endocytosis (Novarino et al., 2010). Together with the experiments with CROPs-deleted TcdA, these results suggest that accelerated endocytosis rate is probably not the only explanation for the enhanced cytotoxicity associated with ClC-5.

### Transport competent and non-transporting CLC-5 similarly support endosomal acidification

**Figure 6:**
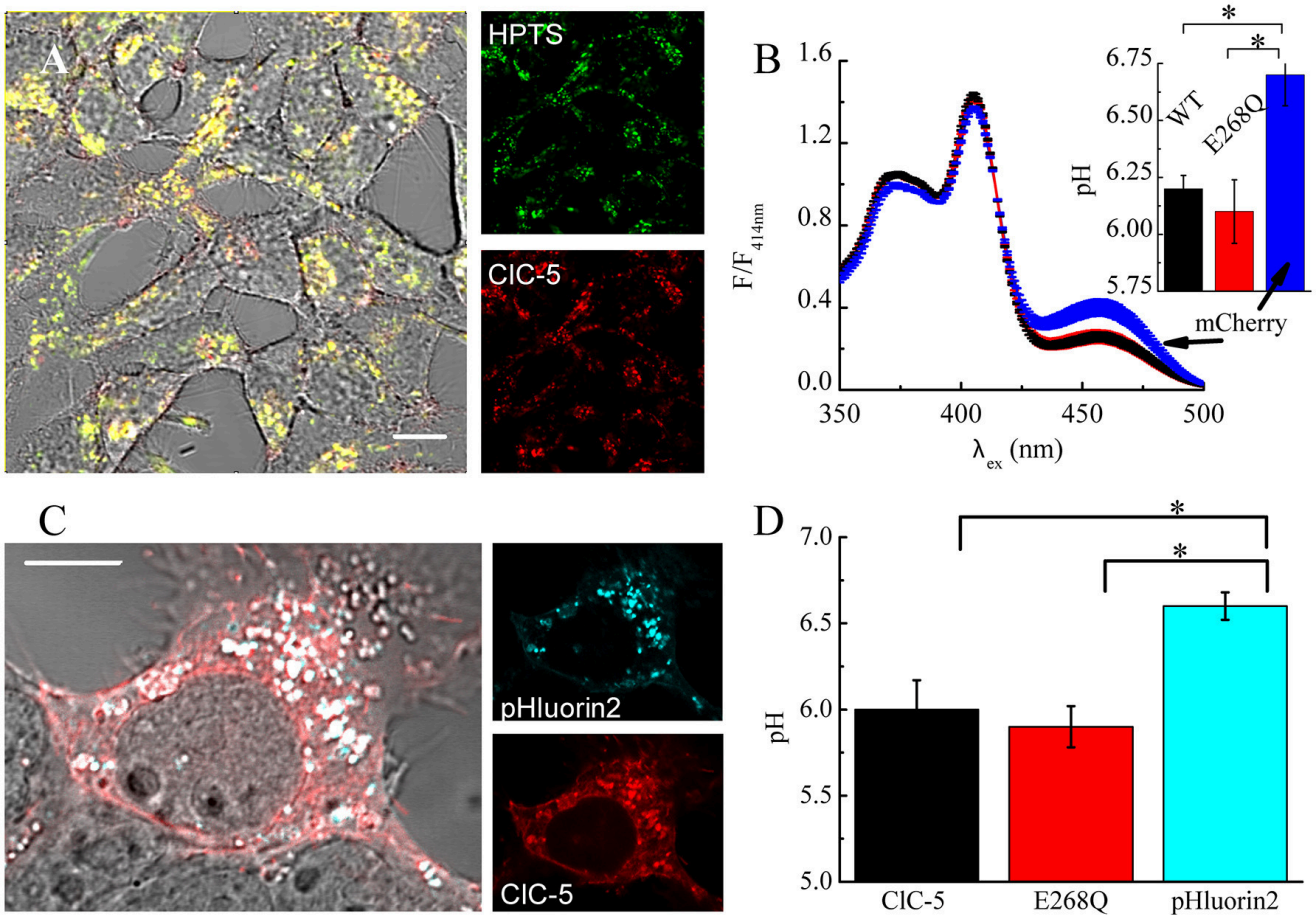
**ClC-5 overexpression promotes endosomal acidification in HEK293 cells. (A)** Localization of endocytosed HPTS dye (right panel, green) and ClC-5 mCherry (right panel, red) in live HEK293 cells, with superimposed fluorescence images in red and green resulting in yellow at colocalization. **(B)** HEK293 cells, stably expressing ClC-5 mCherry, the non-transporting ClC-5 E268Q mCherry, or mCherry alone were incubated with 100 μM HPTS for 24 h and the fluorescence at 510 nm was recorded upon excitation at 350–500 nm in a Horiba Fluorolog spectrophotometer. Intensities are normalized to the isosbestic point at 414 nm. The inset displays the pH obtained by calculating the ratio of the HPTS fluorescence at 510 nm exited at 450 and 390 nm, respectively and converting it to pH using an empirically constructed calibration curve (Supplementary Figure S6). Results are presented as mean ±SEM (*n* = 4, ^*^p < 0.05). **(C)** Representative HEK293T cells transiently transfected with ClC-5 mCherry (red) and synapto-pHluorin2 (green). **(D)** Analysis of vesicular pH of HEK293T cells transiently co-expressing mCherry tagged ClC-5 or ClC-5 E268Q and synapto-pHluorin2 or solely expressing synapto-pHluorin2. The pH was determined using an empirically constructed calibration curve (Supplementary Figure S6). Results are shown as means ±SEM. ^*^Indicates a significant difference between two data sets with *p* < 0.05 (*n* = 46 cells for ClC-5, *n* = 44 for ClC-5 E268Q and *n* = 34 cells for synapto-pHluorin2). Scale bars correspond to 10 μm.

Theoretical considerations by us have shown that the increased membrane capacitance resulting from the expression of E268Q would decrease the energy barrier opposing V-ATPase proton pumping into the endosomal lumen (Guzman et al., 2013). Therefore, we next measured vesicular pH in mock-transfected cells and in cells expressing ClC-5 WT or mutant E268Q. We used the ratiometric pH indicator 8-hydroxypyrene-1,3,6-trisulfonic acid (HPTS) that labels intracellular vesicles upon internalization (Overly et al., 1995). Endocytosed HPTS exhibited a high degree of colocalization with ClC-5 (Figure 6A). The dye is characterized by its dual-peak excitation spectrum with a real pH-independent isosbestic point and a left and right shoulders that become higher and lower upon acidification, respectively (Overly et al., 1995). The HPTS-based measurements showed that vesicular pHs in cells expressing ClC-5 WT and ClC-5 E268Q do not differ from each other and are both significantly lower than the one measured in cells not expressing ClC-5 (Figure 6, Supplementary Figure S8). To additionally test the validity of the results, we performed experiments in which the genetically-encoded vesicular pH reporter synapto-pHluorin2 was used (Miesenböck et al., 1998; Alekov, 2015). These measurements confirmed that vesicular pH in cells expressing the mutant is lower than the one in cells expressing only synapto-pHluorin2 (Figures 6C,D, Supplementary Figure S8). Thus, transport incompetent ClC-5 E268Q is also capable of enhancing vesicular acidification.

Previous experiments by Smith and Lippiat addressed how a similar mutation, ClC-5 E268A, affects endosomal pH. They also found that the endosomal pH of cells expressing the mutant is lower compared to untransfected cells (Smith and Lippiat, 2010a). The smaller magnitude of the observed differences in their experiments could be due to lower expression levels. Moreover, an obligatory colocalization between ClC-5 and the pH probe pHluorin, as implemented here by us, was not imposed in this case (Smith and Lippiat, 2010a). It is possible, therefore, that some of the vesicles analyzed by the authors contained only a small number of ClC-5 transporters. Such effects would lead to an underestimation of the acidifying action ClC-5 E268A.

### ClC-5 is associated with markers of early endosomes in colon epithelial HT29 cells

We next mapped the ClC-5-dependent internalization pathway in HT29 cells by quantifying the colocalization of ClC-5 with proteins of the Rab family that reside in different endosomal subpopulations. In kidney epithelia, ClC-5 is found in the same vesicles as the constitutively active (CA) form of Rab5, a small GTPase residing mainly in early endosomes (Günther et al., 1998).

We, therefore, started our investigations with this variant but also tested the dominant negative (DN) and wild-type (WT) Rab5. Analysis of the confocal images showed that ClC-5 exhibits the highest colocalization with Rab5 CA, a weaker but significant colocalization with Rab5 WT and the lowest colocalization with Rab5 DN (Supplementary Figure S9). Supplementary Figure S10 shows that ClC-5 also colocalizes with the early-to-late endosomal marker Rab7 (Feng, 1995) and with another often-used early endosomal marker Rab4a (Sönnichsen et al., 2000).

Rab4 is known to localize to both the vacuolar and budding compartment of early endosomes (Sönnichsen et al., 2000) whereas Rab5 localizes predominantly to the vacuolar subpopulation (Trischler et al., 1999; Sönnichsen et al., 2000). The colocalization with several Rab proteins suggests that in colon HT29 cells, ClC-5 is localized to a broad subpopulation of intracellular vesicles, including the early (Rab4 and Rab5) and early-to-late endosomes (Rab7). In contrast, very weak colocalization of ClC-5 with the marker for recycling endosomes Rab11 (Ullrich et al., 1996; Sönnichsen et al., 2000) was visible (Supplementary Figure S10).

ClC-5 has been also shown to directly interact with the microtubule motor kinesin2 (Reed et al., 2010). We, therefore, imaged cells expressing both, the microtubule marker EMTB1 and ClC-5. In these cells, some of the ClC-5 containing endosomes appeared in the proximity of stained microtubules (Supplementary Figures S10E,F).

In light of the effects of the non-transporting mutation E268Q ClC-5, we also checked whether mutation E268Q exhibits different localization in HT29 cells (Supplementary Figure S11). However, it showed similar localization as the WT and seems, therefore, to preserve the intracellular localization of ClC-5. In summary, ClC-5 appears to enhance the cytotoxicity of *C. difficile* toxins by accelerating endocytosis and endosomal acidification in a manner that does not depend on its ion transporting capabilities.

## Discussion

The major virulence factors of pathogenic *C. difficile* are two large toxins, TcdA and TcdB, that invade host cells via the endocytotic route and disrupt the gastrointestinal epithelial barrier (Jank et al., 2007; Papatheodorou et al., 2010; Pruitt et al., 2010). The acidification of endocytotic organelles is crucial for the intracellular translocation with subsequent release of the enzymatically active N-terminus into the cytosol (Qa'Dan et al., 2000). We here investigated the role of the anion/proton exchanger ClC-5 for the cytotoxicity caused by TcdA and TcdB. ClC-5 is expressed in gastrointestinal epithelia and is one of the key molecular determinants supporting vesicular acidification along the endocytotic pathway. Our data show that ClC-5 expression dramatically increases the susceptibility to intoxication by both TcdA and TcdB (Figures 1, 4, Supplementary Figure S1). These findings are specific for ClC-5 since such potent effects were not observed (see Figure 1) in cells stably expressing the fluorescent protein mCherry alone, or ClC-4, a closely related intracellular CLC transporter that functions similarly as ClC-5 (Guzman et al., 2013) but is localized mainly in membranes of the endoplasmic reticulum (Okkenhaug et al., 2006). The specificity of the effects and their biological relevance is further demonstrated by the reduced cytopathic effect of TcdA associated with the knockdown of endogenously-expressed ClC-5 (Figure 2). These findings, together with the previously demonstrated physiological role of ClC-5 in human gastric epithelia (Sullivan et al., 2009; Alex et al., 2010; Takahashi et al., 2014), outline ClC-5 as a potential critical part of the host cell machinery attacked and hijacked by *C. difficile* toxins in the processes of cell invasion, cell intoxication and cell killing. Moreover, our findings suggest that the increased expression of ClC-5 associated with IBD (Sullivan et al., 2009; Alex et al., 2010) might be one of the factors leading to the higher incidence, increased severity and higher mortality rates observed upon *C. Difficile* infections (CDI) in patients with IBD (Issa et al., 2008).

At the cellular level, defects of ClC-5 have been demonstrated to impair both receptor-mediated endocytosis and vesicular acidification in mouse kidney (Jentsch, 2008) and in human cells (Gorvin et al., 2013). We performed several additional experiments to test how these two effects enhance the action of *C. difficile* toxins. Firstly, we investigated the effects of ClC-5 on intoxication by TcdA with ablated CROPs domain (TcdA 1-1874, Figure 4). It is known that TcdA can enter cells via different endocytosis routes that are differentially affected when the CROPs domain is removed (Papatheodorou et al., 2010; Olling et al., 2011; Gerhard et al., 2013). The general increase in susceptibility due to ClC-5 observed for TcdA, TcdB and for the CROPs-truncated variants (Figures 1, 4) suggests that a selectively enhanced internalization via one of these pathways is probably not the only mechanism behind these effects. This notion is supported by the fact that ClC-5 expression increases the cytotoxicity of TcdA applied at both the basolateral and the apical side of polarized epithelial cells (Supplementary Figure S2A). In addition, we showed that ClC-5-mediated enhancement of the TcdB cytopathic effects is reversed by preincubation with bafilomycin A1 (Figure 1) that does not block the initial endocytotic steps (Yoshimori et al., 1991; Bayer et al., 1998). This observation outlines the importance of vesicular acidification for the increased susceptibility induced by ClC-5. Thirdly, quantifying the rate of endocytosis showed that it is accelerated to a higher extent by the expression of ClC-5 WT than by the expression of ClC-5 E268Q (Figure 5). In contrast, the cytopathic action of *C. difficile* toxins was enhanced to a similar extent for the two ClC-5 constructs (Figure 4). Finally, the intravesicular pH is lowered to the same extent in cells expressing ClC-5 WT or the non-transporting ClC-5 E268Q. These results represent compelling evidence that ClC-5 increases the susceptibility to TcdA and TcdB by lowering the pH of the early endosomes and by promoting their maturation. Interfering with this process seems, therefore, a promising strategy for treating or preventing CDI. Unfortunately, proton pump inhibitors have been shown to increase the risk of CDI, probably due to inhibitory effects on the growth of the normal bacterial gut flora (Sharma et al., 1984; Jonkers et al., 1996; Howell et al., 2010; Janarthanan et al., 2012). The identification of ClC-5 as a mammalian protein potentially involved in the internalization and activation of *C. difficile* toxins underlines, therefore, the need for discovering, characterizing and testing specific inhibitors or modulators of this transporter in gastrointestinal epithelia.

An additional important finding reported here is the ability of mutant ClC-5 E268Q to support vesicular acidification and accelerate intoxication by TcdA and TcdB to a similar extent as ClC-5 WT (Figures 4, 6). To our knowledge, this is the first report describing the physiological impact of this non-transporting ClC-5 mutant. In addition to abolishing electrogenic transport, mutation E268Q also increases the electric membrane capacitance (Grieschat and Alekov, 2012). As such, it is capable of reducing the positive electric potential build up by the proton pumping action of the V-ATPases, i.e., it allows more protons to be transported into the vesicular lumen in expense for the same amount of hydrolyzed ATP (Guzman et al., 2013). The capacitive currents accompanying the activation of mammalian CLC transporters seem, therefore, to be not merely a byproduct of their voltage-dependent gating. In contrast, they might serve distinct and important physiological functions, similar to the capacitance alternations associated with the gating of neuronal cation channels (Hodgkin, 1975). These results suggest that the invasion and processing of bacterial toxins directly depend on the physical properties of the endosomal membrane. Accordingly, age and developmental changes are to be expected. Of note, reduced risk of CDI is observed in patients taking statins, but not other cholesterol-lowering drugs (Naggie et al., 2011; Motzkus-Feagans et al., 2012). As cholesterol is well known to increase the electric capacitance of the lipid bilayer, the aforementioned effects might be due to a reduced acidity of endosomal compartments in these patients. It appears, therefore, interesting to elucidate whether statins alter more potently the cholesterol content of endosomal membranes than of the plasma membrane and whether endosomal pH is indeed altered upon statin treatment.

The ability to support vesicular acidification was recently reported for an analogous non-transporting mutant in another CLC transporter, ClC-7, that regulates acid secretion in the ruffled border of bone-resorbing osteoclasts (Weinert et al., 2014). It is also interesting that transfecting mutant E268Q ClC-5 enhances toxin internalization to a lesser extent compared to WT ClC-5. A physiologically relevant link between intravesicular Cl^−^ content and endocytosis has been established previously (Novarino et al., 2010). Therefore, the lower toxin uptake (when compared to the uptake in cells expressing ClC-5 WT) might be attributed to an alteration of the intravesicular Cl^−^ concentration that originates from the non-transporting phenotype of mutant E268Q. Investigating if the corresponding mutant in ClC-7 also increases the membrane capacitance might clarify whether its pathophysiological impact (Weinert et al., 2014) also relies on effects resembling the ones described here by us. Electrogenic ClC-5 transport has been shown to supports the very initial V-ATPase-independent vesicle acidification, immediately after their formation. The underlying mechanism seems to be direct ClC-5 proton transport into the vesicular lumen coupled to the extrusion of Cl^−^. (Smith and Lippiat, 2010a) The similarly high intoxication rates observed for cells expressing transport-competent and incompetent ClC-5 suggests, therefore, that not the very initial endocytotic steps but accelerated endosomal maturation underlies the enhanced effects of *C. difficile* toxins.

In summary, our findings provide strong evidence that host intracellular ion transporters, and in particular ClC-5, facilitate the processing of *C. difficile* toxins and enhance thereby their cytopathic action. These novel insights into the cellular role of ClC-5 and its ability to enhance the virulence of *C. difficile* toxins in gastrointestinal cells reveal additional molecular targets at the host side that are potentially hijacked by pathogen bacteria and widen the horizon of possibilities for much-needed therapies of life-threatening gastrointestinal bacterial infections.

## Materials and methods

### Constructs, cell culture, and protein expression

The assembly of the used pRcCMV ClC-4 and pRcCMV ClC-5 constructs is described in detail previously (Hebeisen et al., 2003; Grieschat and Alekov, 2012; Guzman et al., 2013). For creating stable ClC-5-expressing MDCK II cells under blasticidin selection, the DNA fragment coding for ClC-5-mCherry was subcloned into the pCDNA6 vector by PCR. The mRFP-Rab5 construct was kindly provided by Ari Helenius (Addgene plasmid #14437) (Vonderheit and Helenius, 2005). The Rab5 mutants mCherry-Rab5DN (S34N) and mCherry-Rab5CA (Q79L) were provided by Sergio Grinstein (Addgene plasmid #35139 and #35138) (Bohdanowicz et al., 2012). EGFP-Rab4a was kindly provided by Marino Zerial (University of Technology, Dresden) (Vitale et al., 1998). EGFP-Rab11 WT and EGFP-Rab7 WT were provided by Richard Pagano (Addgene plasmid #12674 and #12605) (Choudhury et al., 2002) and the construct EMTB-3xGFP by William Bement (Addgene plasmid#26741) (Miller and Bement, 2009). The synapto-pHluorin2 construct was kindly provided by Dr. Raul Guzman (FZ Jülich, Jülich, Germany) and used as described previously (Alekov, 2015).

HT29 cells (ATCC #HTB-38) were cultivated in full medium containing Dulbecco's MEM/ Hams F-12 (Biochrom), 50 units/ml penicillin/streptomycin (Invitrogen) and 10% FBS (Gibco) and transfected using the jetPRIME transfection reagent (Polyplus Transfection) according to the manufacturer's protocol. HEK293T cells (ATCC #CRL-3216) were maintained in DMEM (Gibco), supplemented with 10% FBS (Gibco), 2 mM L-glutamine and 50 units/ml penicillin/streptomycin (Invitrogen) and transfected using calcium phosphate precipitation (Graham and van der Eb, 1973). HEK293 cells (ATCC #CRL-1573), permanently expressing mCherry-tagged ClC-5 WT or ClC-5 E268Q and ClC-4 and mCherry alone, as well as MDCK II cells (ATCC #CRL-2936) permanently expressing mCherry-tagged ClC-5 WT, were created as described previously (Hebeisen et al., 2003) and cultivated in minimum Eagle's medium (MEM) (Gibco), supplemented with 10% FBS (Gibco) and 900 μg/ml Geneticin (G418, Invitrogen) or 1 μg/ml blasticidin for MDCK II cells. HEK293T cells were transfected using the calcium phosphate precipitation method. For transiently transfected cells, fluorescence imaging was performed 24–48 h after transfection.

### Expression, purification, and labeling of TcdA

TcdA and TcdB, as well as their truncated forms and fusion proteins, were recombinantly expressed as 6xHis-tagged proteins using the *Bacillus megaterium* expression system as described before (Burger et al., 2003). The TcdA-EGFP fusion was generated as previously described by Olling et al. by fusion of TcdA 1-1874 and the N-terminally EGFP tagged CROPs (aa 1,875–2,710) (Olling et al., 2011). All Proteins were purified by gravity flow by Ni-affinity chromatography using Ni-TED columns (Macherey & Nagel) following the standard protocol supplied by the manufacturer. The specific protein concentration of the toxins was estimated by SDS-PAGE. To fluorescently label the primary amino groups of TcdA, the Atto425 Protein Labeling Kit (Jena Bioscience GmbH) was used following the manufacturer's protocol.

### *C. difficile* toxin internalization and cytopathic effects associated with cell morphology

To investigate the internalization and localization of the major *C. difficile* toxins, HT29, HEK293T and HEK293 cells were grown on glass coverslips coated with Poly-L-lysine (Sigma-Aldrich). 300 μL of Leibovitz L-15 medium (Gibco) containing 200 nM EGFP-tagged TcdA was added to the cells and the mixture was incubated on ice for 30 min during which the toxins bind to the cells without being internalized. Cellular uptake of the toxin was initiated by transferring the cell/toxin mixture to 37°C or room temperature. At indicated time points, the reaction was stopped by removing the toxin-containing solution and washing with ice-cold PBS (Gibco). For live-cell imaging, the coverslips containing the intoxicated cells were transferred in Tyrode's solution (150 mM NaCl, 5 mM KCl, 1 mM MgCl_2_, 10 mM HEPES, and pH 7.4) and imaged on a spinning disc confocal microscope (see below). Alternatively, cells were fixed using methanol (10 min at −20°C) or 4% PFA diluted in PBS (15 min at RT), washed three times with PBS and mounted on a microscope slide with 15–20 μL of Fluoromount-G medium (SouthernBiotech). Fixed cells were imaged using a laser scanning Zeiss LSM780 microscope (see below).

Endocytosis rate and vesicular acidification capacity of ClC-4 and ClC-5 transformed HEK293 cells was estimated by limited uptake of TcdB. Abrogation of toxin uptake was achieved by bafilomycin A1-mediated inhibition of v-ATPase. To this end, cells were treated with high concentration of TcdB (2 nM in cell culture medium) to saturate surface receptors and toxin uptake was allowed for 10 min. Then, pH-dependent translocation process of TcdB from endosomes to cytosol was terminated by addition of bafilomycin A1 (100 nM). The translocation capacity of cells was estimated by quantification of rounded cells after further 90 min incubation. Proof of bafilomycin A1-mediated inhibition of TcdB effects was given by 10 min preincubation of cells before application of TcdB. Maximum cytopathic effect (cell rounding) was shown by incubation of cells with 2 nM TcdB alone.

To measure the amount of internalized TcdA, the primary amino groups of full-length TcdA were labeled using the Atto425 Protein Labeling Kit (Jena Bioscience GmbH) according to the manufacturer's protocol. HEK293 cells stably expressing mCherry, ClC-5 mCherry or ClC-5 E268Q mCherry were incubated on ice in Leibovitz medium for 15 min. Then the medium was replaced by Leibovitz L-15 medium containing 200 nM TcdA and incubated on ice for 45 min. To allow toxin internalization, the cells were transferred to 37°C for 5 min and the unbound toxin was washed off three times with PBS (Gibco). Finally, cells were resuspended in 2 mL Tyrode solution and the fluorescence of the labeled TcdA was analyzed at 425 nm excitation and an emission of 440–650 nm in a Horiba Fluorolog spectrophotometer. Afterward, the absorbance of the samples was measured at 560 nm using a Victor3 multilabel reader. To determine the number of cells in the sample, calibration curves for the absorbance at 560 nm for each cell line were performed. Subsequently, cells were separated using 0.25% Trypsin-EDTA (Gibco) and resuspended in medium. After a centrifugation step at 300 × g for 3 min, cells were washed once with PBS (Gibco) and centrifuged again (3 min at 300 × g). Subsequently, cells were resuspended in 3 ml Tyrode solution and counted using a Neubauer chamber.

For the cytotoxicity assay, cells were seeded in 24-well chambers and grown for 24 h to sub-confluence. Toxins were diluted in cell culture medium at various concentrations and added to the cells. Toxin-induced cell rounding was monitored by light microscopy and the cytopathic effect was quantified as the percentage of rounded cells compared to the total cell number.

For all experiments with a direct comparison between effects, toxin from the same preparation batch was used to avoid possible activity differences.

### ClC-5 siRNA knockdown

To reduce the levels of ClC-5 in HT29 and HEK293 cells, the ON-TARGETplus Human CLCN5 (1,184) siRNA SMART pool (Dharmacon, #L-006153-00-0005) was used. One day before transfection, cells were seeded on glass pre-coated with Poly-L-lysine (Sigma-Aldrich). 10 nM and 20 nM of siRNA were transfected. For microscopic analysis of cells intoxicated with TcdA, a plasmid caring the coding sequence of the leucocyte antigen CD-8 was cotransfected. To verify the knockdown of ClC-5, RNA was extracted using the Isol-RNA Lysis Reagent (5PRIME). Cells were scraped off the plate and washed once with PBS. Then Isol-RNA Lysis Reagent was added and RNA was extracted according to the manufacturer's protocol. The integrity of the RNA was checked on an 2% agarose gel and its concentration was measured using a Nanodrop Photometer (Thermo Fischer Scientific). To remove genomic DNA contamination, a DNase I digestion was performed using the RQ1 RNase-Free DNase (Promega) according to the protocol. cDNA was generated with the AMV First Strand cDNA Synthesis Kit (New England BioLabs). Finally, the level of ClC-5 mRNA was analyzed using appropriate forward (GGGGCTATTTGGGTAAGTGGACTC) and reverse primers (GCAAAGAATGAACGCCACAAT). Expression levels were normalized to the housekeeping gene phosphoglycerate kinase 1 (PGK1) (Jacobsen et al., 2014). For PGK1, the forward (GAGATGATTATTGGTGGTGGAA) and reverse primers (AGTCAACAGGCAAGGTAATC) were used. A quantitative RT-PCR was carried out using PowerUP SYBR Green Master Mix (Thermo Fisher Scientific) in triplicates with ClC-5 and PGK1 primers. The expression levels of ClC-5 were normalized to PGK1 levels and the 2^−ΔΔCT^ was calculated (Livak and Schmittgen, 2001). To validate the knockdown not only on the mRNA level but also on the protein level, HEK293 cells stably transfected with mCherry-tagged ClC-5 were transfected with the siRNA and after 72 h the fluorescence of the ClC-5-mCherry protein was investigated.

To analyze the cytopathic effect of TcdA on HT29 cells with reduced levels of ClC-5, cells were incubated with 3 nM TcdA (3 h at 37°C) 72 h after the transfection, and the percentage of rounded cells to cells with healthy morphology was calculated. To identify HT29 cells with a high probability of transfection with siRNA, cells were cotransfected with a plasmid encoding the CD8 antigen and incubated before use with polystyrene microbeads pre-coated with anti-CD8 antibodies (Dynabeads M-450 CD8, Dynal) (Jurman et al., 1994). Only cells decorated with microbeads (see Supplementary Figure S3C) were then analyzed for rounding. In a blinded control test, the rounding of cells carrying beads was analyzed in parallel by J. Bleidorn. The outcome of the test was identical, excluding possible bias introduced by the investigator.

### Measurement of transepithelial electrical resistance (TER)

TER measurements were performed as described previously (Gerhard et al., 1998). In brief, cells (untransfected or stably transfected with mCherry-tagged ClC-5) were seeded onto filter inserts (Nunc) at a density of 10^5^ cells per cm^2^. Cells were then grown for 5 days to build a differentiated monolayer. The TER was measured using an EVOM volt/ohm meter equipped with an EndOhm chamber (World Precision Instruments). Only cell layers with an initial resistance of >75 Ω × cm^2^ were used for experiments.

### Fluorescence microscopy and colocalization analysis

Live cells were imaged on an Olympus *BX61WI* microscope equipped with a Yokogawa *CSU-XI* spinning disc unit. mCherry was excited at 561 nm, EYFP at 515 nm, EGFP and the Atto425 labeling dye at 445 nm. Fluorescence was detected using a filter set with a GFP emitter of 537/26 nm (also used for YFP detection) and mCherry emitter of 609/54 nm. A Zeiss LSM 780 confocal laser scanning microscope was used for imaging of fixed cells and for the measurement of endosomal pH in living cells. EGFP was excited at 488 nm and mCherry at 561 nm. Emission was recorded between 493 and 574 nm (EGFP) as well as 578–696 nm (mCherry). For testing the localization of HPTS, the dye was excited at 405 nm and emission was detected at 490–520 nm together with mCherry excited at 561 nm and 578–696 nm. Synapto-pHluorin2 was excited at 405 nm and 488 nm; emission was recorded at 494–574 nm. The autofluorescence intensity of untransfected and untreated cells was determined in each of the channels and used to ensure the specific detection of the transfected proteins or internalized fluorescent labels.

For colocalization analysis, dual color fluorescence images were subjected to linear spectral unmixing (Zimmermann, 2005). For the object based colocalization analysis, the Fiji plugin SQUASSH was used (Rizk et al., 2014). The following optimized parameters were applied: “background removal” true, “window size” 10, “regularization” 0.05, “min intensity ch1” 0.05, “min intensity ch2” 0.05, “subpixel” true, “cell mask ch1” true, “mask threshold ch1” 0.0145 for fixed cells (0.0011 for Atto425 labeled toxin and 0.0059 for live cell images of GFP-tagged TcdA), “cell mask ch2” true, “mask threshold ch2” 0.0049 (0.001 for Atto425 labeled toxin and 0.0072 for live cell images of GFP-tagged TcdA), “intensity estimation automatic noise model” Poisson. Parameters used for analyzing cells expressing ClC-5 or ClC-5 E268Q cotransfected with various Rab variants: “background removal” true, “window size” 10, “regularization” 0.05, “min intensity ch1” 0.15, “min intensity ch2” 0.15, “subpixel” true, “cell mask ch1” true, “mask threshold ch1” 0.0066–, “cell mask ch2” true, “mask threshold ch2” 0.0049, “intensity estimation automatic noise model” Poisson. 3D Z-stacks were used for all colocalization analysis.

### SDS-PAGE of whole cell lysate and immunoblotting

Twenty four hour after transfection, cells were washed twice with PBS, scraped off the Petri dishes and resolved in 1 mL PBS. Cells were then washed by centrifugation (10 s, 100 × g) and resuspended in 1 mL PBS. Finally, the pellet was dissolved in 100 μL lysis buffer (150 mM NaCl, 10 mM HEPES, 1% Triton, 1% vol. Complete Protease Inhibitor Cocktail (Roche, freshly added), pH 7.5). The suspension was incubated on ice for 45 min with a 10-s vortex step every 5 min followed by a centrifugation step for 15 min at 13,000 rpm and 4°C. The supernatant was transferred into a new collection tube and quantified using a Bradford assay (Bradford, 1976). The samples were analyzed by Laemmli gel electrophoresis with 10% SDS running gel layered with a 4% stacking gel (Laemmli, 1970). The bands of fluorescence-tagged ClC-5 and the used protein ladder (Spectra Multicolor Broad Range Protein Ladder, Thermo Scientific) were imaged with a fluorescence scanner (FUSION SL, Vilber Lourat). Intracellular glucosylation of substrate GTPases by TcdA or TcdB was quantified by Western blot using glucosylation-sensitive monoclonal Rac1-antibody clone 102 (Transduction Laboratories BD) (Genth et al., 2006). For graphical analysis, Western blot signal intensity was analyzed densitometrically. Rac1 signals were normalized to the Rac1 signals of the untreated samples. Beta-actin was used as housekeeping protein and marker for total protein load and detected with a monoclonal antibody (clone AC15, Sigma-Aldrich).

### Measurement of vesicular pH

For HPTS-based measurements of vesicular pH, HEK293 cells permanently expressing mCherry-tagged ClC-5 WT, mutation E268Q or mCherry alone were incubated for 24 h in culture medium supplemented with 100 μM HPTS (AnaSpec, Inc). Cells were rinsed twice with PBS, separated using 0.25% Trypsin-EDTA (Gibco) and washed (centrifugation for 3 min at 1,200 rpm, removal of supernatant and resuspension of the pellet) once in medium and twice in PBS to remove the extracellular dye. After the final washing step, cells were resuspended in Tyrode's solution and the fluorescence of internalized HPTS was measured in a Horiba Fluorolog spectrophotometer. Excitation spectra from 350 to 500 nm were recorded at 510 nm emission. To generate a calibration curve, HEK293T cells were separated and washed as described above. Then the cells were resuspended in hypoosmotic buffer (150 mOsm, Tyrode solution diluted 1:2) containing 0.5 mM HPTS. The mixture was then incubated at 37°C for 1 h. Afterward, the cells were washed 3 times with buffers having a pH ranging from pH 5 to 8 (15 mM MES/HEPES, 130 mM KCl). For the last washing step (centrifugation for 3 min at 300 × g), pH Buffer containing 10 μM nigericin (A.G. Scientific, Inc.) was used and the mixture was incubated for 10 min before the HPTS fluorescence was measured. Intensities were normalized to the isosbestic point at 414 nm.

The measurement of vesicular pH in HEK293T cells transiently co-expressing synapto-pHluorin2 and mCherry-tagged WT or E268Q ClC-5, as well as the construction of a calibration curve, were conducted as previously described (Alekov, 2015). In brief, cells were illuminated at 405 and 488 nm to excite synapto-pHluorin2 and at 561 nm for mCherry with emission ranges of 494–574 nm and 578–696 nm. Vesicular regions were selected using semi-automated analysis in the ClC-5 channel (when co-expressed) or in one of the pHluorin2 channels for measuring the vesicular pH of cells transfected only with the pH-reporter construct. The fluorescence ratio F_488_/F_405_ was calculated and converted to absolute pH using an experimentally determined calibration curve. For the latter, cells transiently expressing synapto-pHluorin2 were bathed in Tyrode's solutions containing 10 μM nigericin (A.G. Scientific, Inc.) with different acidities and illuminated at 488 and 405 nm. The fluorescence from membrane regions in the two excitation channels was used to calculate the ratio F_488_/F_405_.

### Data analysis and statistics

Graphical visualization and statistics of endpoints for evaluation of toxic effects (cell rounding, Rac1-glucosylation) were performed with GraphPad Prism 5.02 (GraphPad Software). The rest of the data were analyzed and graphed using Excel (Microsoft), Origin (MicroCal), and MatLab (MathWorks).

## Authors contributions

FR, AO, RA, DR, MG, RG, and AA performed the experiments and analyzed the data. AO, RG, AA designed the research, analyzed the data. AZ analyzed the data. FR, RG, and AA wrote the paper. All authors revised the manuscript.

### Conflict of interest statement

The authors declare that the research was conducted in the absence of any commercial or financial relationships that could be construed as a potential conflict of interest.
