# Supplementary material for: Overexpression of the Endosomal Anion/Proton Exchanger ClC-5 Increases Cell Susceptibility toward Clostridium difficile Toxins TcdA and TcdB
Source: Front Cell Infect Microbiol. 2017 Mar 13;7:67. doi: 10.3389/fcimb.2017.00067 (PMC5346576; doi:10.3389/fcimb.2017.00067)
Supplement: Supplementary file 1 [file Presentation1.PDF]

Supplementary Material

Overexpression of the Endosomal Anion/Proton Exchanger ClC-5 Increases Cell Susceptibility towards *Clostridium Difficile* Toxins TcdA and TcdB

Frederike Ruhe[1], Alexandra Olling[2], Rasmus Abromeit[1], Dennis Rataj[2], Matthias Grieschat[1], Andre Zeug[1], Ralf Gerhard[2]*, Alexi Alekov[1]*

[1]Institute for Neurophysiology, Hannover Medical School, D-30625 Hannover, Germany

[2]Institute for Toxicology, Hannover Medical School, D-30625 Hannover, Germany

*** Correspondence:**

A. K. Alekov Email: alekov.alexi@MH-Hannover.de, alexi.alekov@gmail.com

Institute for Neurophysiology, Hannover Medical School, Carl-Neuberg-Str. 1, D-30625 Hannover, Germany, Tel: ++49 511 532 9391; Fax: ++49 511 532 2776

R. Gerhard Email: gerhard.ralf@MH-Hannover.de

Institute for Toxicology, Hannover Medical School, Carl-Neuberg-Str. 1, D-30625 Hannover, Germany, Tel: ++49 511 532 2810; Fax: ++49 511 532 2879

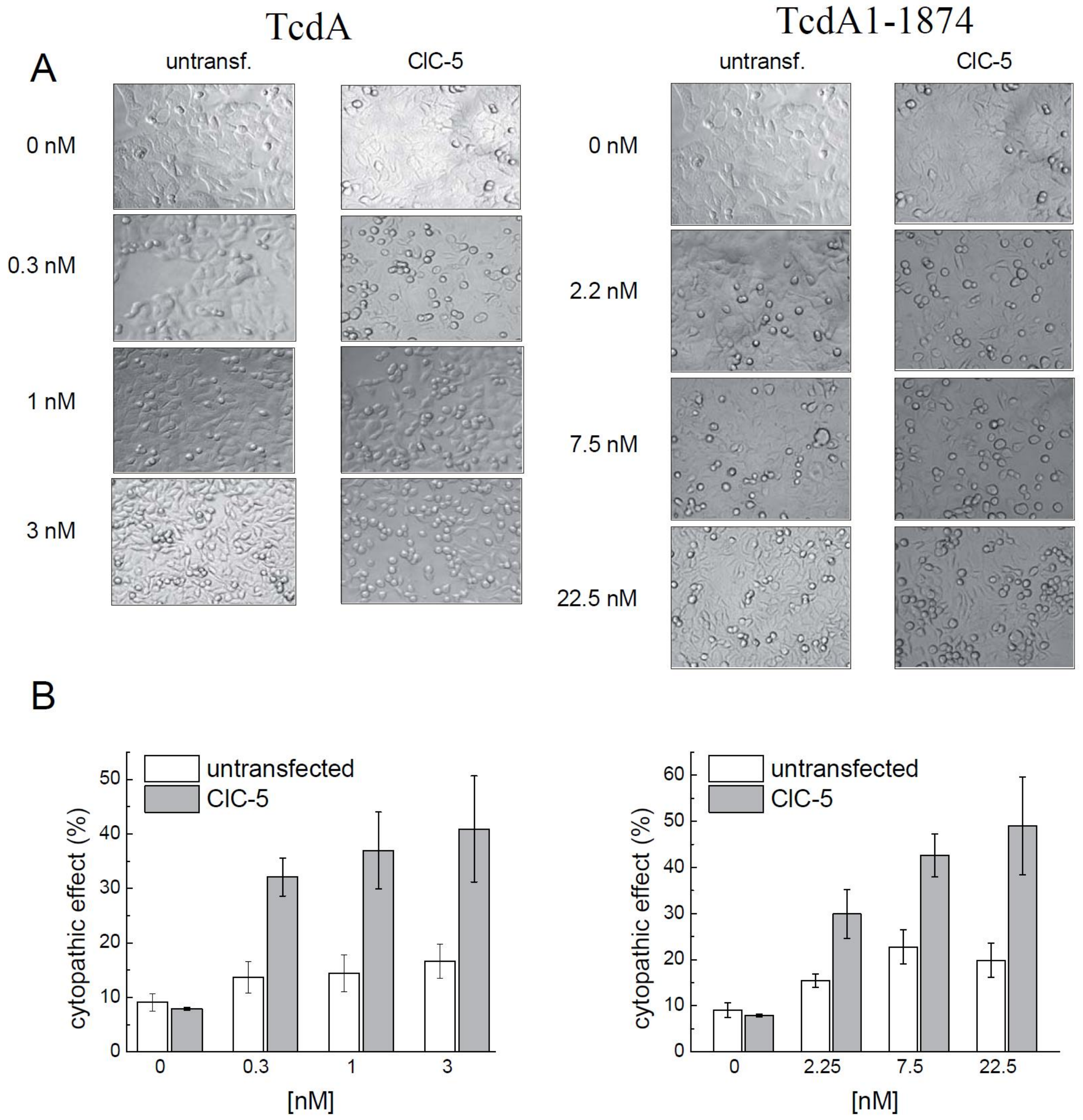

Supplementary Figure S1. Increased TcdA susceptibility of ClC-5-transfected HT29 cells. A) HT29 cells (untransfected and transfected with ClC-5-mCherry) were treated with various concentrations of full-length TcdA (left) or CROP-truncated (right) TcdA 1-1874. The micrographs depict cell rounding induced by toxin-catalyzed glucosylation of Rac1 GTPases. **B)** Concentration-dependent cytopathic effect of TcdA or TcdA 1-1874 on untransfected and ClC-5-mCherry expressing HT29 cells (mean ±SD are depicted, n=3 experiments), measured by calculating the proportion of roundish cells to cells with healthy morphology.

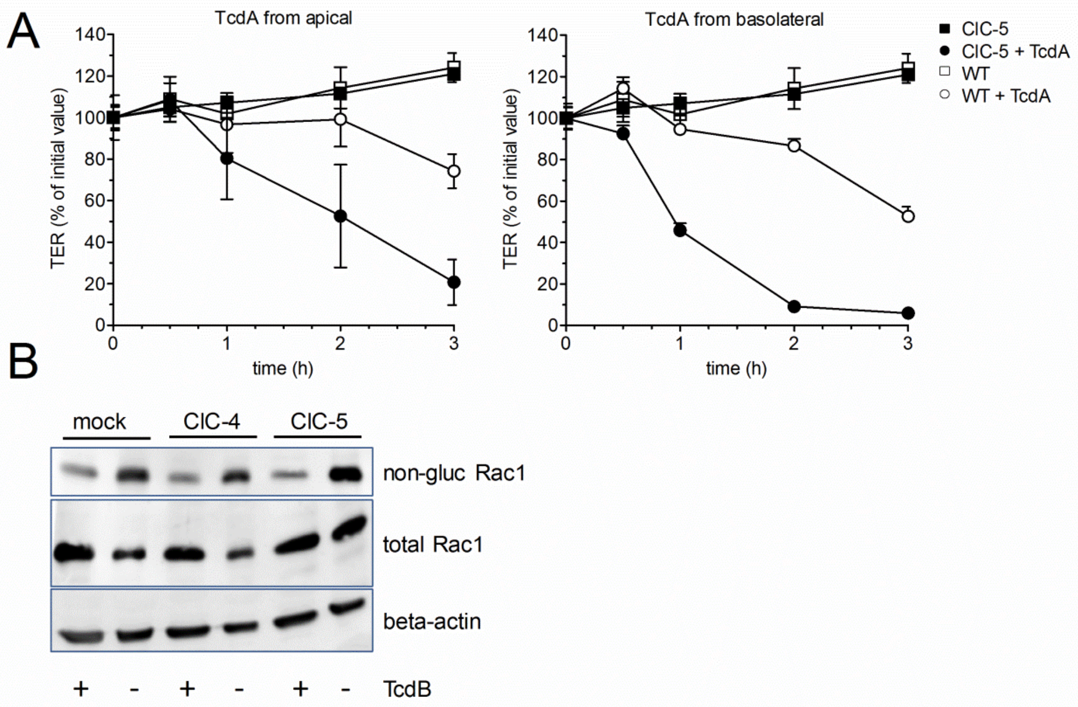

Supplementary Figure S2. Time course of toxin-induced effects in MDCK II cells and evaluation of total Rac1 and non-glucosylated Rac1 in HEK293 cells. A) To demonstrate the time course of the toxin-induced effects, we monitored the transepithelial electrical resistance (TER) of MDCK II monolayers of cells cultivated to confluency on filter inserts. TcdA (1.5 nM) was applied from the apical or basolateral side of untransfected MDCK II cells (WT) or MDCK II cells stably expressing ClC-5 and the TER was measured using an EVOM volt/ohm meter at defined time points. The data of three independent experiments are summarized as mean values ± SD. **B)** Stably transfected HEK293 cells (with ClC-4, ClC-5, and mCherry alone - mock) were treated with 0.3 nM TcdB for 2 h. Glucosylation of Rac1 was detected using glucosylation-sensitive monoclonal Rac1/Cdc42 antibody (clone 102, BD Transduction laboratories). The total Rac1 level was similarly detected (antibody clone 23A8, Upstate Biology).

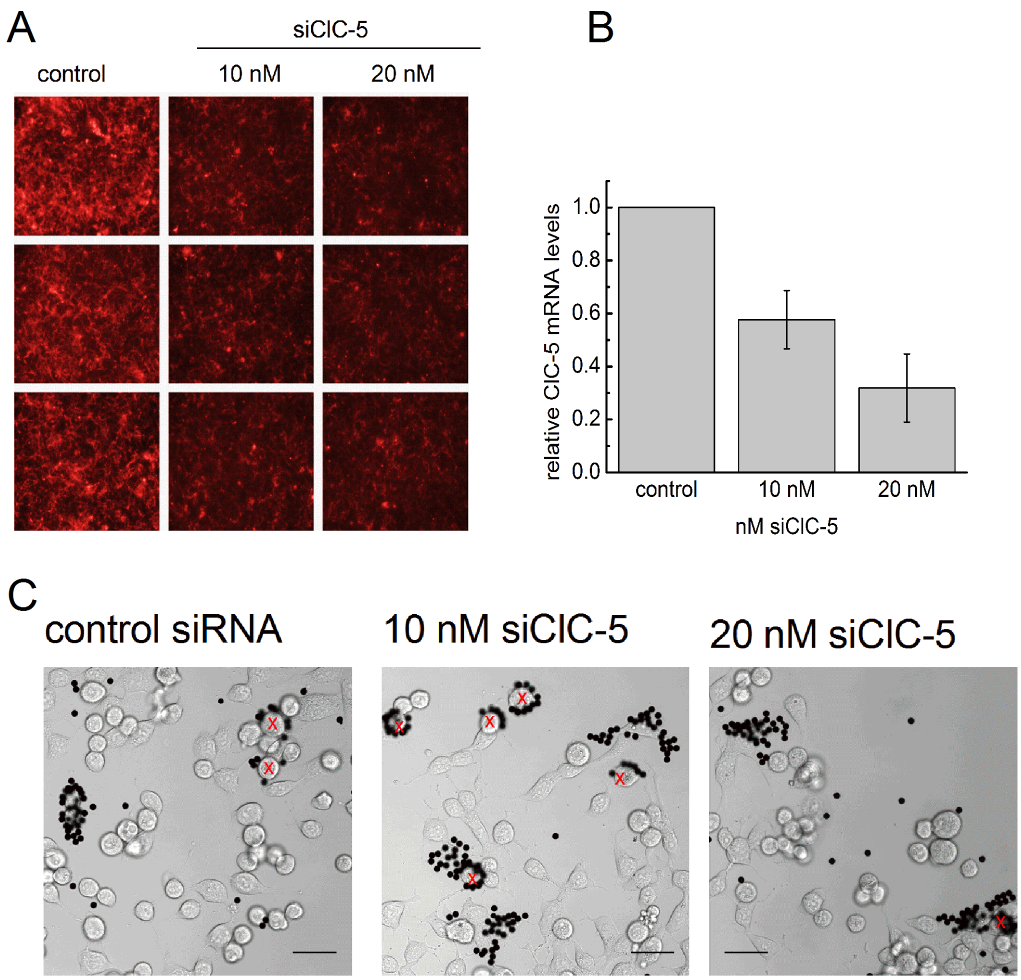

Supplementary Figure S3. Knockdown of ClC-5 by small interfering RNA (siRNA). ClC-5 was knocked down by siRNA targeted against human ClC-5 (siClC-5). As a negative control, non-targeting siRNA was used. **A)** Representative fluorescence images of HEK293 cells stably expressing ClC-5 mCherry. Cells were treated with 10 or 20 nM siClC-5 for 72 h. The downregulation of ClC-5 is visualized by the reduced mCherry fluorescence intensity. Three separate experiments are depicted. **B)** Quantitative RT-PCR for HT29 cells transiently transfected with 20 nM control siRNA or 10 and 20 nM siClC-5. Relative ClC-5 mRNA levels were measured against PGK1 and normalized to the value of the control cells (see Materials and Methods). Bars represent mean values ± SEM of 3 independent experiments. **C)** Representative images demonstrating the use of microbeads (black dots) coated with anti-CD8 antibodies to identify cells with a high probability of being transfected with siRNA (see Materials and Methods). HT29 cells transfected with non-targeting siRNA (control) or 10 and 20 nM siClC-5 were intoxicated with 3 nM TcdA for 3 h at 37°C. To distinguish between transfected and nontransfected cells, a plasmid containing the CD8 sequence was cotransfected and cells were incubated with the beads. Only cells decorated with beads (more than two to exclude accidental colocalization) were analyzed. Cells that were considered “rounded” are marked with a red x. Scale bars correspond to 40 µm.

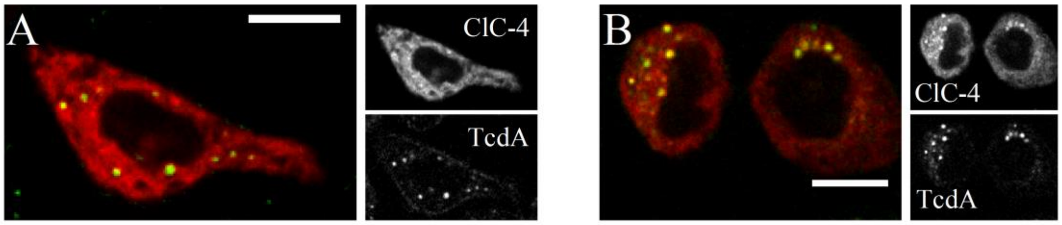

Supplementary Figure S4. Colocalization of TcdA-EGFP and ClC-4 in living cells. Localization of TcdA-EGFP and ClC-4 mCherry in HT29 living cells. **A and B)** Representative cells transiently expressing mCherry-tagged ClC-4 (red) were incubated with TcdA-EGFP (green) for an interval of 5 - 20 min at RT (n=9). Single z-slices were taken on a spinning disc confocal microscope. Scale bars correspond to 10 µm.

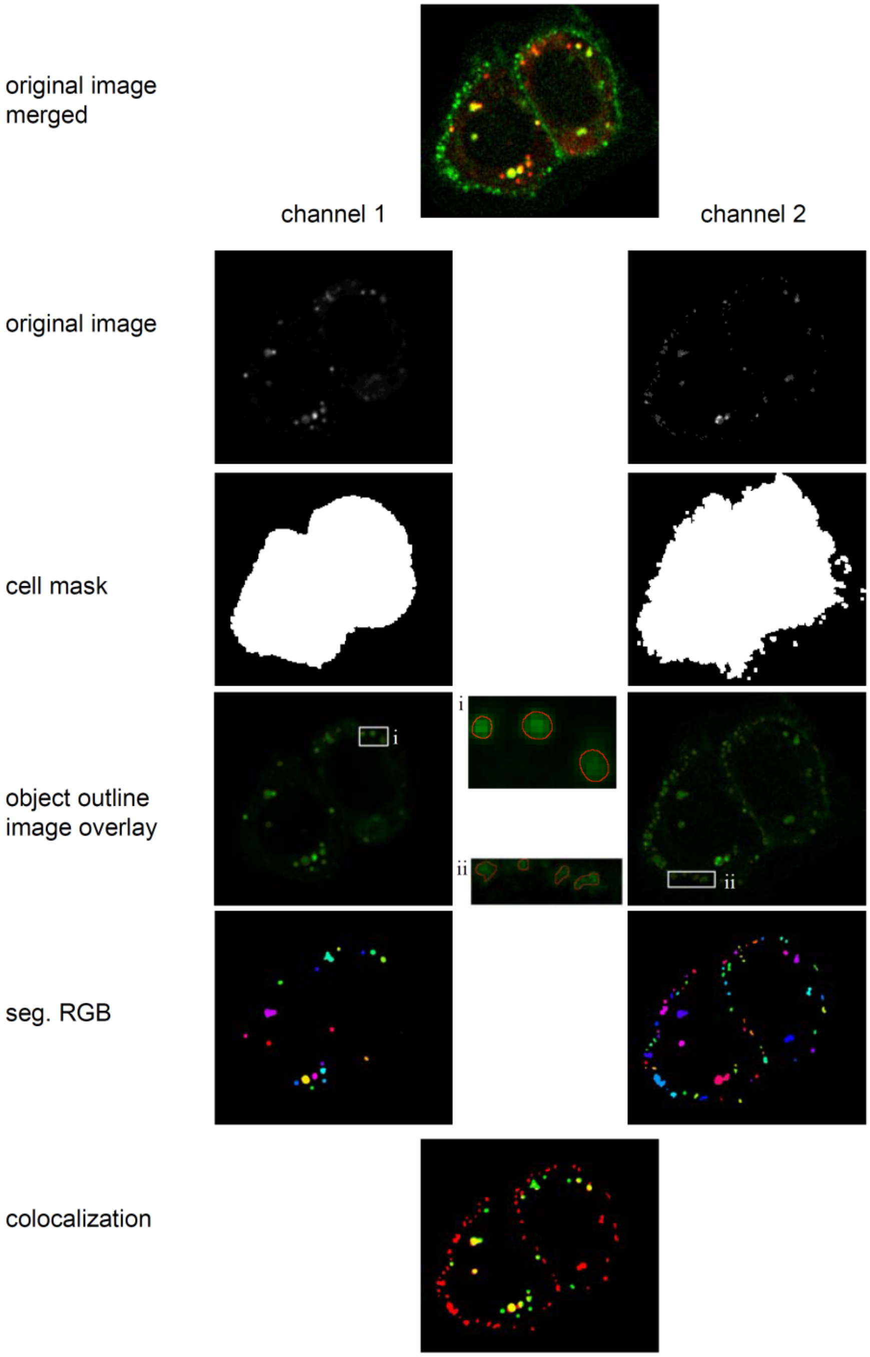

Supplementary Figure S5. Colocalization workflow for the used Fiji plugin SQUASSH. Composite image showing the overlay of dual color confocal images of mCherry-tagged ClC-5 (in red as original image channel 1) and TcdA-EGFP (in green as original image channel 2). The" cell mask" shows the identified cell area set by thresholding and hole-filling. The 'object outline image overlay' represents an overlay of the original image with the segmented objects outlined in red. "Seg. RGB" visualizes each object in a different, random color. 'Colocalization' denotes the overlay of objects from both channels. The FIJI plugin SQUASSH calculates from the identified regions the number of overlapping objects between the two channels (object-based colocalization) as well as the overlapping area between the channels (size-based colocalization).

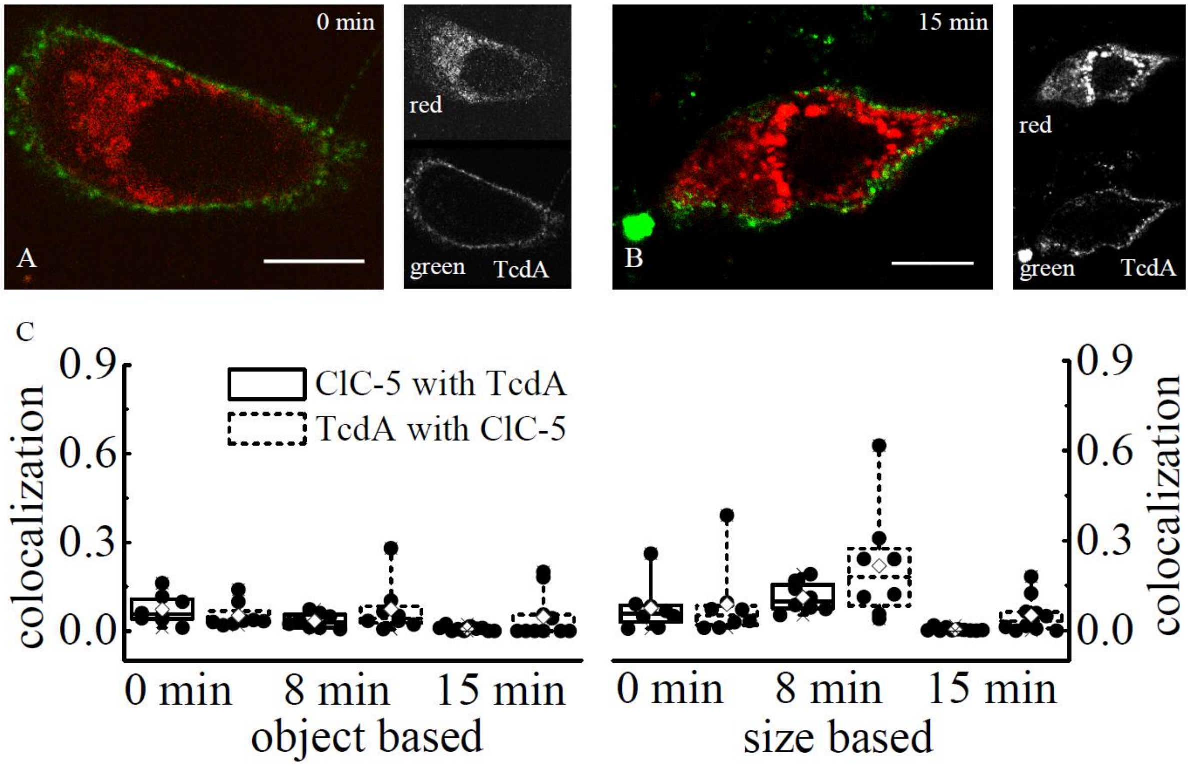

Supplementary Figure S6. Localization of TcdA-EGFP and ClC-5 in fixed HT29 cells. A), B) Representative images of fixed HT29 cells transfected with ClC-5 mCherry with internalized EGFP labeled TcdA obtained using a Zeiss LSM 780 confocal laser scanning microscope. Cells transiently expressing mCherry-tagged ClC-5 (red) were fixed immediately upon toxin (TcdA-EGFP in green) incubation at 4°C (A) and upon additional 15-min incubation at 37°C to allow toxin internalization (B). **C)** Box plot summarizing the results from an object (left) and size based (right) colocalization analysis (8 cells for 0 and 8 min and 10 cells for 15 min of toxin internalization were analyzed, respectively; single cells (●), 25 – 75 % (☐, large box), mean (◊) as well as 99 % and 1 % respectively (X)) corresponding to the number of overlapping objects and the fraction of overlapping objects defined in two channels as ClC-5 and TcdA containing objects. Scale bars correspond to 10 µm.

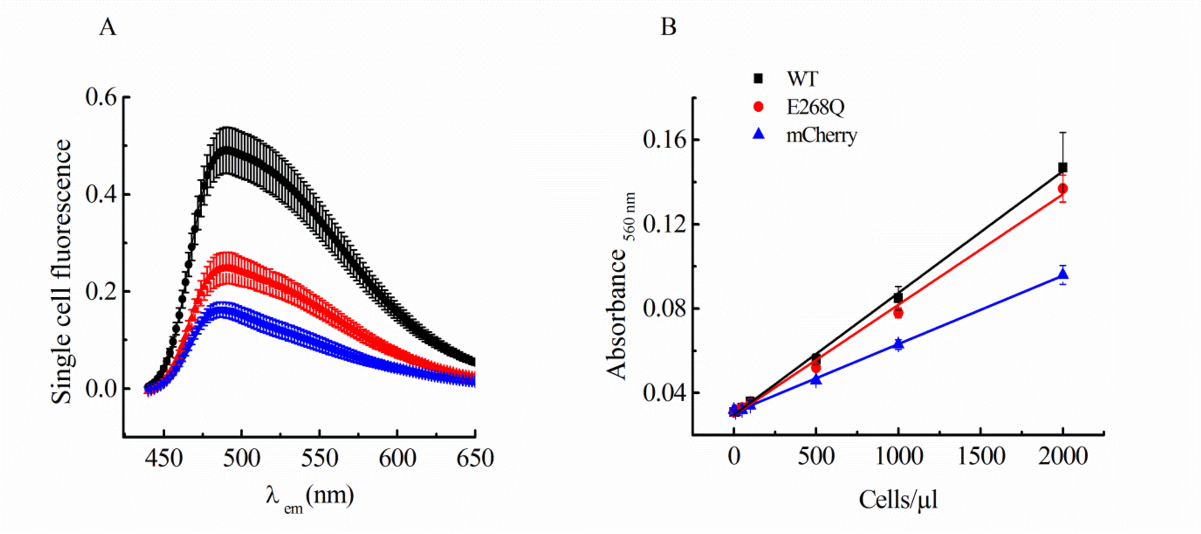

Supplementary Figure S7. Fluorescence intensity per cell of endocytosed TcdA in HEK293 cells stably overexpressing mCherry or mCherry tagged ClC-5 or ClC-5 E268Q. A) HEK293 cells stably transfected with the fluorescent mCherry (blue), ClC-5-mCherry (WT, black) and ClC-5-mCherry E268Q (E268Q, red) were incubated for 5 min at 37°C with 200 nM TcdA, covalently labeled with Atto425. Fluorescence of the labeled TcdA was analyzed at 425 nm excitation and an emission of 440-650 nm in a Horiba Fluorolog spectrophotometer. The absorbance of the samples was measured at 560 nm using a Victor3 multilabel reader. The fluorescence per cell was thus calculated using calibration curves for each cell line (see (B)). 9 Petri dishes were analyzed for ClC-5 WT and E268Q and 10 for mCherry on two different days. Means ±SEM are shown. **B)** Calibration curves for correlating cell absorbance at 560 nm and cell number/µm. HEK293 cells stably transfected with fluorescent mCherry (blue), ClC-5-mCherry (black) and ClC-5 E268Q mCherry (red) were counted using a Neubauer chamber. Cells were then diluted in Tyrode's solution and the absorbance at 560 nm was measured using a Victor3 plate reader. Two different experiments for each cell line were performed with 3 repetitions each. The results shown are means ±SEM.

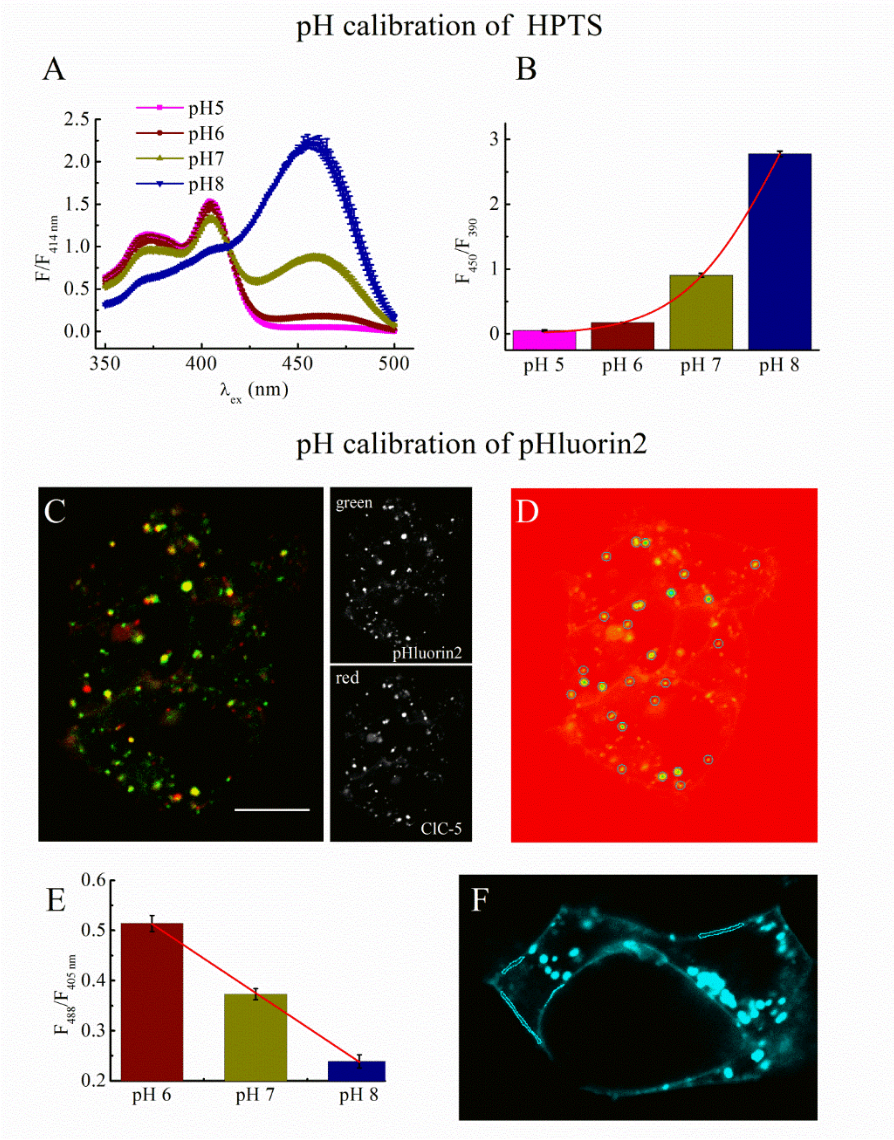

Supplementary Figure S8. Calibration of the pH-measurement in HEK293T cells. As a pH indicator, either HPTS (A and B) or pHluorin2 (C-F) was used. **A)** The cell cytosol of HEK293T cells was loaded with HPTS by incubation in a hypoosmotic solution containing 0.5 mM HPTS for 1 h. Cells were washed and resuspended in buffers with different pH ranging from pH 5 - pH 8. The pH buffers contained 10 µM nigericin to equilibrate intracellular and extracellular pH. The fluorescence at 510 nm was recorded upon excitation at 350-500 nm in a Horiba Fluorolog spectrophotometer. Intensities are normalized to the isosbestic point at 414 nm (n = 2). **B)** Calibration curve of HPTS of F_{450}/F_{390} (ratio of the HPTS fluorescence at 510 nm excited at 450 nm and 390 nm) as a function of pH. **C)** Localization of ClC-5 mCherry (red) and pHluorin2 (green) in HEK293T cells. Images were taken with a Zeiss LSM 760 confocal microscope with mCherry being excited at 561 nm and synapto-pHluorin2 at 405 and 488 nm. **D)** Representative image of vesicles (encircled) detected by the analysis software. Particles in the mCherry channel were detected and the fluorescence intensity ratio F_{488}/F_{405} was calculated. **E)** Calibration curve of synapto-pHluorin2 (n = 11 cells for pH 6, n = 10 for pH 7 and n = 9 for pH 8). For the calibration curve, HEK293T cells transiently expressing synapto-pHluorin2 were maintained in a solution of specific pH containing 10 µM nigericin. Stained plasma membrane regions were chosen to calculate the F_{488}/F_{405} ratio as depicted in **F)**.

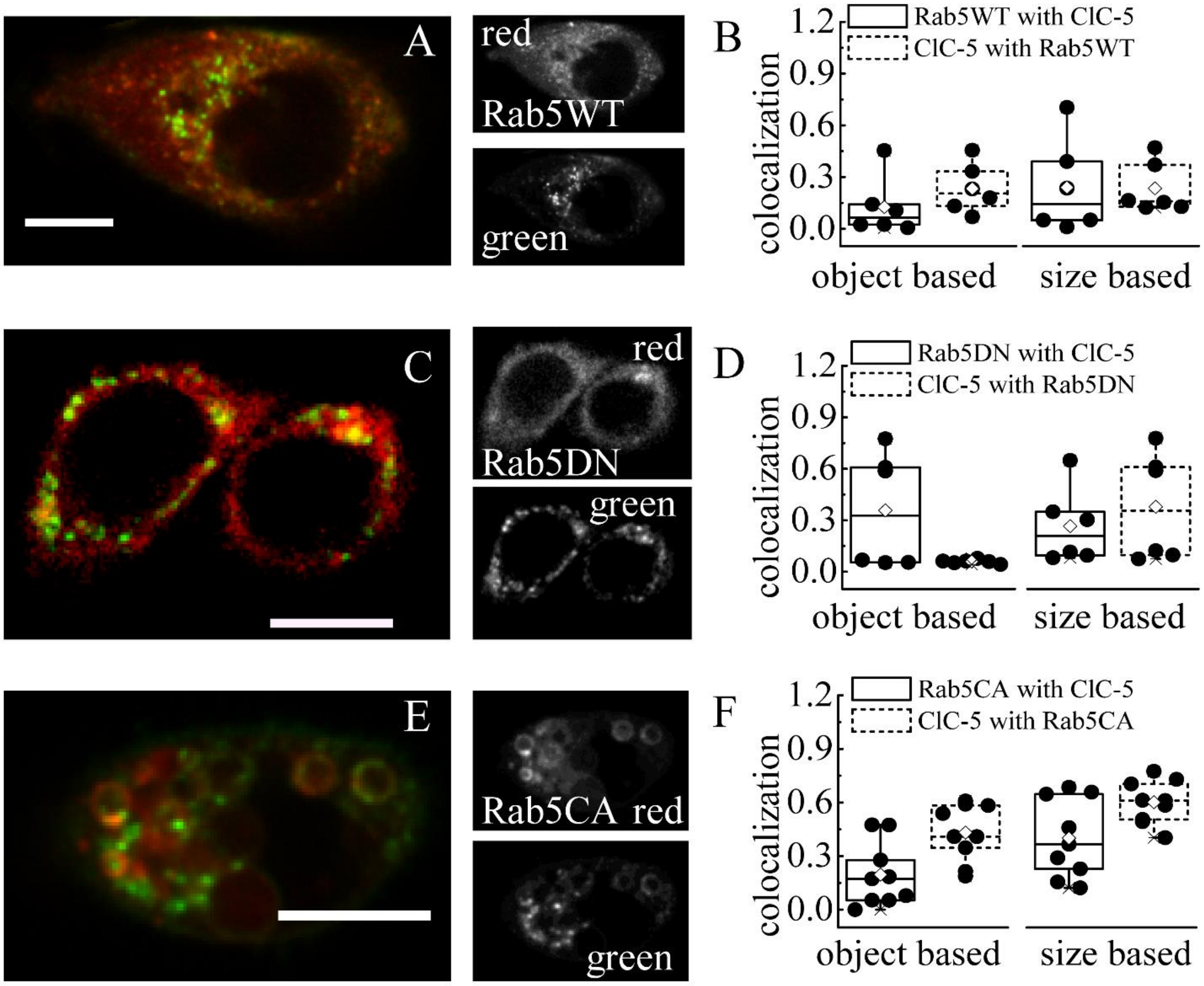

Supplementary Figure S9. Colocalization of ClC-5 with Rab5 (Rab5WT), and a dominant negative (Rab5DN) and constitutively active (Rab5CA) mutants of Rab5. **A), C), E)** Representative images from a spinning disc confocal microscope showing the localization of ClC-5-EYFP (in green) together with either Rab5WT-RFP (A), Rab5DN-mCherry (C) or Rab5CA-mCherry (E) (all three Rab5s in red) in HT29 cells. **B), D), F)** Box plot summarizing the results from an object (left) and size based (right) colocalization analysis corresponding to the number of overlapping objects and the fraction of overlapping objects defined in two channels as Rab5WT-, Rab5Dn-, Rab5CA- and ClC-5-containing objects as depicted in (A), (C) and (E). Six cells for the RabWT and Rab5DN as well as 9 cells for Rab5CA cotransfections were analyzed (single cells (●), 25 – 75 % (☐, large box), mean (◊), median (—) as well as 99 % and 1 % (X)). Scale bars correspond to 10 µm.

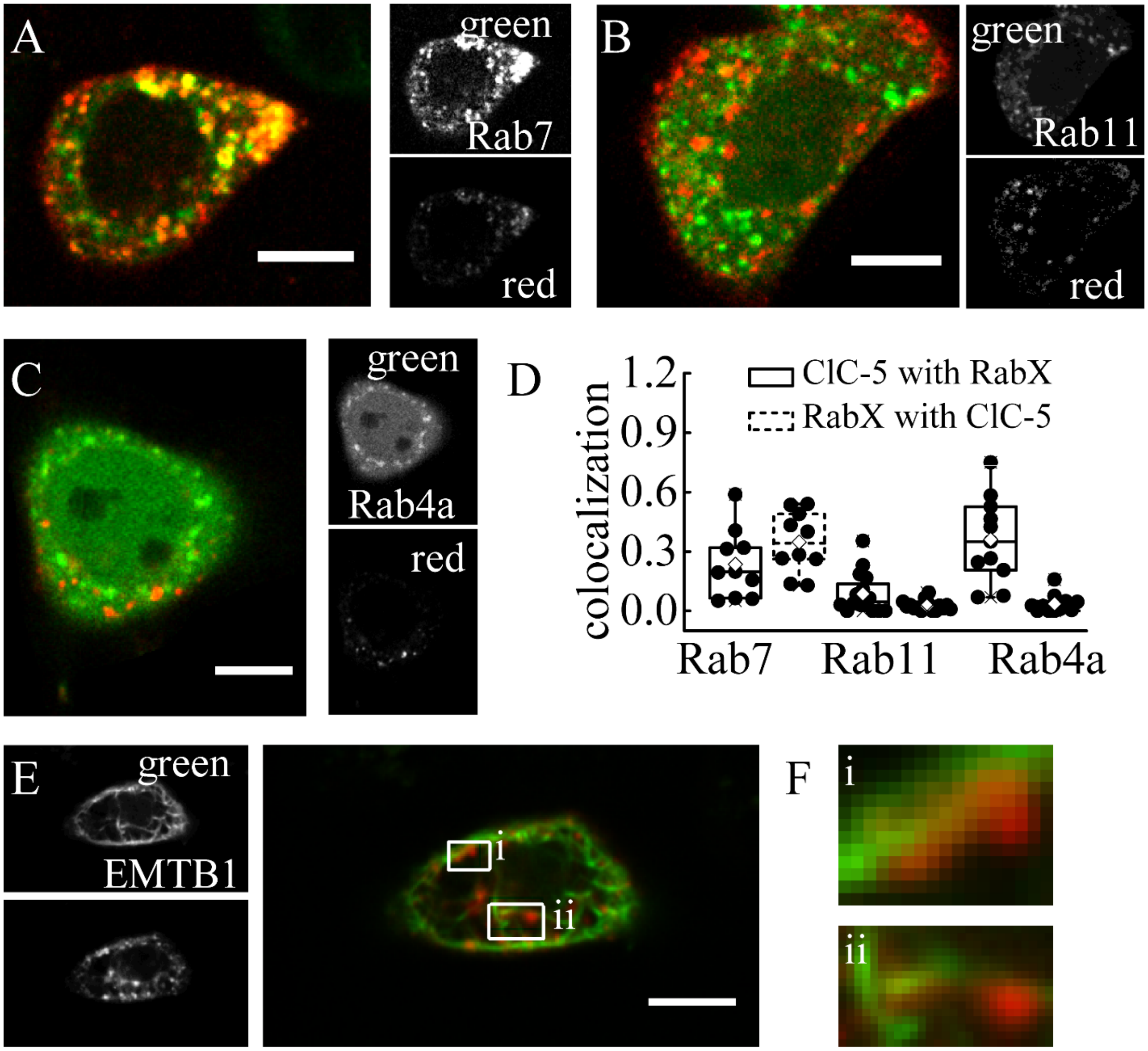

Supplementary Figure S10. Colocalization of ClC-5 with Rab7, Rab11, and Rab4a as well as association with EMTB1. A)-C) Representative confocal images showing HT29 cells cotransfected with ClC-5-mCherry (red) and either Rab7-EGFP, Rab11-EGFP or Rab4a-EGFP (all in green), respectively. **D)** Box plot summarizing the results from an object based colocalization analysis corresponding to the number of overlapping objects defined in two channels as Rab7-, Rab11- or Rab4a- and ClC-5- containing objects (10 cells for Rab7 were analyzed as well as 11 cells for Rab4a and 16 cells for Rab11; single cells (●), 25 – 75 % (□, large box), mean (◊), median (─) as well as 99 % and 1 % (X)) **E and F)** Representative confocal image showing the localization of GFP-tagged microtubule marker EMTB1 (green) and ClC-5-mCherry (red). Scale bars correspond to 10 µm. All images were obtained using a spinning disc confocal microscope.

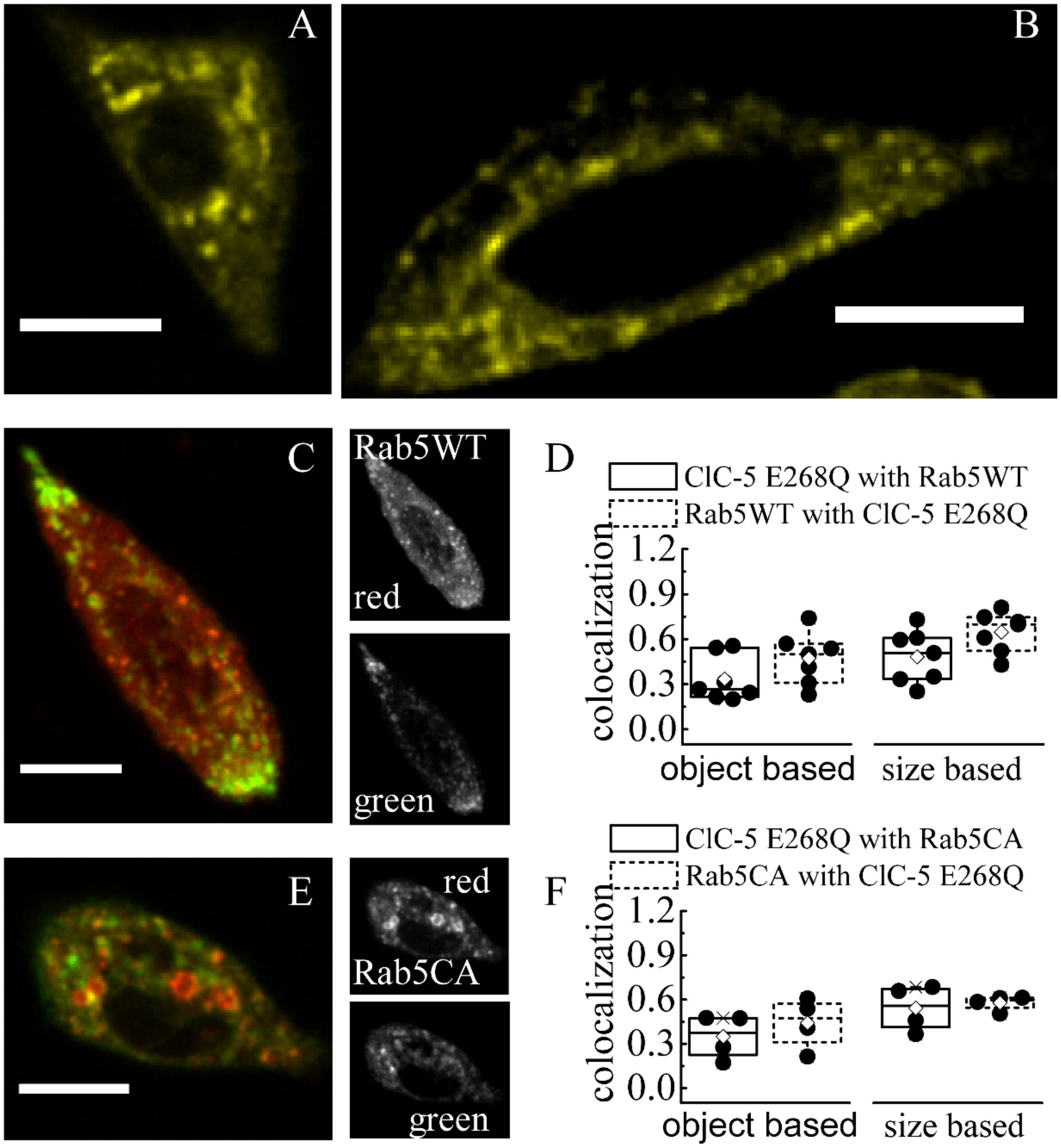

Supplementary Figure S11. Colocalization of the non-transporting mutant ClC-5 E268Q with Rab5 (Rab5WT) and a constitutively active (Rab5CA) mutant of Rab5. **A), B)** Representative confocal images obtained using a spinning disc confocal microscope from HT29 cells transfected with ClC-5-EYFP E268Q. **C), E)** Representative confocal images showing the localization of ClC-5-EYFP E268Q (in green) together with either Rab5WT-RFP (C) or Rab5CA-mCherry (E) (both Rab5s in red) in HT29 cells. Single z-slides are shown in all images. **D, F)** Box plots summarizing the results from object-based and size-based colocalization analysis corresponding to the number of overlapping objects defined in two channels as Rab5WT- or Rab5CA- and ClC-5-containing objects (7 cells for Rab5WT and 4 cells for Rab5CA were analyzed; single cells (●), 25 – 75 % (□, large box), mean (◊), median (─) as well as 99 % and 1 % (X)). Scale bars correspond to 10 µm.
